# Climate and Ecosystem Factors Mediate Soil Freeze‐Thaw Cycles at the Continental Scale

**DOI:** 10.1029/2024JG008009

**Published:** 2024-11-27

**Authors:** Erin C. Rooney, Angela R. Possinger

**Affiliations:** ^1^ National Soil Survey Center USDA‐NRCS Lincoln NE USA; ^2^ Earth and Planetary Sciences University of Tennessee Knoxville Knoxville TN USA; ^3^ School of Plant and Environmental Sciences Blacksburg VA USA

**Keywords:** freeze‐thaw, climate, NEON, soil temperature, sensor, biome

## Abstract

Freeze‐thaw cycles (FTC) alter soil function through changes to physical organization of the soil matrix and biogeochemical processes. Understanding how dynamic climate and soil properties influence FTC may enable better prediction of ecosystem response to changing climate patterns. In this study, we quantified FTC occurrence and frequency across 40 National Ecological Observatory Network (NEON) sites. We used site mean annual precipitation (MAP) and mean annual temperature (MAT) to define warm and wet, warm and dry, and cold and dry climate groupings. Site and soil properties, including MAT, MAP, maximum‐minimum temperature difference, aridity index, precipitation as snow (PAS), and organic mat thickness, were used to characterize climate groups and investigate relationships between site properties and FTC occurrence and frequency. Ecosystem‐specific drivers of FTC provided insight into potential changes to FTC dynamics with climate warming. Warm and dry sites had the most FTC, driven by rapid diurnal FTC close to the soil surface in winter. Cold and dry sites were characterized by fewer, but longer‐duration FTC, which mainly occurred in spring and increased in number with higher organic mat thickness (Spearman's *⍴* = 0.97, *p* < 0.01). The influence of PAS and MAT on the occurrence of FTC depended on climate group (binomial model interaction *p* (χ^2^) < 0.05), highlighting the role of a persistent snowpack in buffering soil temperature fluctuations. Integrating ecosystem type and season‐specific FTC patterns identified here into predictive models may increase predictive accuracy for dynamic system response to climate change.

## Key Points

- Freeze‐thaw drivers differed across sites grouped by annual temperature and precipitation regime
- Warm and dry sites had the highest freeze‐thaw frequencies due to diurnal winter temperature fluctuations
- The influence of air temperature on freeze‐thaw was dependent on integrative ecosystem characteristics of climate groups

## 1 Introduction

Critical ecosystem functions respond to changes in temperature and precipitation (IPCC, 2019). Climate change‐driven disturbances modify above and belowground processes that regulate vegetation productivity, soil functions, and greenhouse gas emissions through changes to the biological, chemical, and physical properties of the soil. In addition to direct impacts of climate, the interaction of dynamic climate and site factors with soil properties can create conditions for physical and biogeochemical disturbance of the soil environment. Increasing or decreasing frequency of freeze‐thaw cycles (FTC) can shift nutrient dynamics and alter structure‐development processes across a range of soil systems, with an outsized impact on SOC climate vulnerability (Henry, 2007; Lipson & Schmidt, 2004; Figure S1 in Supporting Information S1).

Freeze‐thaw cycles are a seasonal form of soil disturbance that occur in a variety of landscapes including desert, prairie, forest, and tundra (Chen et al., 2020; Henry, 2007; Wang & Bettany, 1993; Wang et al., 2015). FTC can alter key soil functions such as SOC stability through reorganization of soil physical structure (e.g., water retention, soil stability, and erosion) and changes in biogeochemical cycling (e.g., microbial biomass turnover, and nutrient fluxes) (Freppaz et al., 2007; Inamdar et al., 2018; Liu et al., 2021; Patel et al., 2021; Rooney, Bailey, Patel, Dragila, et al., 2022; Rooney, Bailey, Patel, Possinger, et al., 2022; Schimel & Clein, 1996; Xu et al., 2022; Yang et al., 2022; Zhang et al., 2021). The occurrence of FTC in a given soil depends on interactions among climate conditions such as air temperature, precipitation, and snow cover, as well as landscape properties such as organic soil thickness, vegetation structure, and canopy (Decker et al., 2003; Fu et al., 2017; Xu et al., 2022). The interaction of these conditions and their correlation to FTC frequency is sometimes unique to an ecosystem or biome (Chen et al., 2020; Guo et al., 2018; Inamdar et al., 2018; Liu et al., 2016). Although previous work has investigated the potential biogeochemical impacts of freeze‐thaw at a global scale (Song et al., 2017), the drivers of freeze‐thaw have generally been documented in isolation within a given ecosystem type or biome, and relative importance of controls on FTC across biomes has been rarely studied. As air temperature, precipitation, snowpack, and organic matter dynamics shift under global climate change (IPCC, 2023; Wieder et al., 2019), understanding the relative importance of these controls is critical for the prediction of future FTC occurrence and frequency across biomes.

Climatic and ecosystem factors that control FTC both modify and collectively interact with incoming solar radiation and sensible heat transfer at the soil surface (Gao et al., 2010; Wang et al., 2019). Solar elevation, latitude, surface albedo, aspect, and slope interact to determine incoming radiation (Kumar et al., 1997; Seyednasrollah & Kumar, 2019). Air and soil temperatures (determined by geographical area and presence of permafrost) decide temperature gradient, which controls sensible heat flux at the soil surface (Sauer & Horton, 2005). Snowpack insulates soil, reducing heat energy transfer into or out of the soil surface (positive or negative sensible heat flux, respectively), as documented in alpine meadow, farmland, permafrost tundra, and forest systems (Campbell et al., 2010; Chen et al., 2020; Wang et al., 2019; Xu et al., 2022). The influence of snow cover on soil temperature can depend on ecosystem and mean annual air temperature (Euskirchen et la., 2007; Lawrence & Slater, 2010; Yi et al., 2015) as well as seasonality of snowpack and the presence of permafrost (Zhang, 2005). At the Hubbard Brook Experimental Forest (HBEF) in the Northeastern United States, snowpack dynamics influence soil temperature variability, where both decreased snow water equivalent (SWE) of the snowpack (associated with warming air temperatures) and decreased snow depth resulted in an increasing depth of soil freezing due to the loss of soil insulation against winter temperatures (Hardy et al., 2001; Wilson et al., 2020), resulting in disruption of the physical soil structure (Groffman et al., 2001a, 2001b). Increased snowpack in Arctic soils was correlated with an increase in thaw and warming, especially in deeper soils below 50 cm (Yi et al., 2015), with variability in the timing and depth of soil fall freeze linked to snow cover variability (Yi et al., 2019). The influence of interacting factors of warming air temperatures and changing precipitation on freeze‐thaw cycle frequency is highlighted by soil temperature data provided by the National Resource Conservation Service (NRCS) at Toolik Field Station on Alaska's north slope where there is a visible 16‐year trend in increasing depth and frequency of FTC in tundra soils (Figure S1 in Supporting Information S1; Soil Survey Staff, Natural Resources Conservation Service, United States Department of Agriculture, 2020). Interactions among air and soil temperatures, snowpack effects, and FTC occurrence underscore the importance of linking these dynamic ecosystem factors to predict how future FTC (and associated impacts to soil function, especially SOC cycling) may vary across biomes.

The impact of snow and soil type on radiation exposure and heat conduction at the soil surface differs across sites as a function of contrasting vegetation or other site properties (Idso et al., 1975; Pomeroy et al., 2009; Romanovsky et al., 2010; Sauer & Horton, 2005). In tundra and forest systems, vegetation structure interacts with winter precipitation and wind to influence air and soil temperatures and thus freeze‐thaw via snow accumulation, sublimation, and ablation (Guo et al., 2018; Mott et al., 2018). Vegetation structure and organic surface soils can further drive biome and site‐level differences in freeze‐thaw by decreasing incoming solar radiation and failing to buffer soils against air temperature fluctuations (Chen et al., 2020; Hedstrom & Pomeroy, 1998; Pomeroy et al., 2009; Shur & Jorgenson, 2007; Strasser et al., 2011). In cultivated systems within the cold, temperate Heilongjiang Province of northeastern China, the absence of vegetation cover increased freeze‐thaw by failing to buffer against air temperature (Xu et al., 2022). In contrast, vegetation canopy (including mosses and lichen) within forest and grassland landscapes combined with surface peat layers to lessen the impact of rising temperatures from 1980 to 2015, resulting in lower soil temperatures with less sensitivity to climate conditions (Xu et al., 2022). Similar influences of vegetation on soil temperature were reported in both the discontinuous and continuous permafrost region of Alaska. Spruce canopy was found to reduce winter soil insulation against colder temperatures by lessening snowpack and enhancing summer insulation against warm air temperatures through moss understory and thick peat horizons at the soil surface (Shur & Jorgenson, 2007). Organic surface soils can further influence freeze‐thaw, with low bulk density organic soil horizons providing ground insulation against summer air temperature (Ahmed et al., 2021; Shur & Jorgenson, 2007). The dynamic interactions of site‐specific vegetation with climate and soil properties on FTC frequency are further complicated by seasonality as well as the complexity of changing winter conditions and cold weather over the past decades with climate change (Contosta et al., 2019). The dominant season in which freeze‐thaw occurs can vary among ecosystems with unique responses of soil respiration and carbon flux across multiple biomes (Arndt et al., 2020; Contosta et al., 2019; Liu et al., 2016).

**Figure 1:**
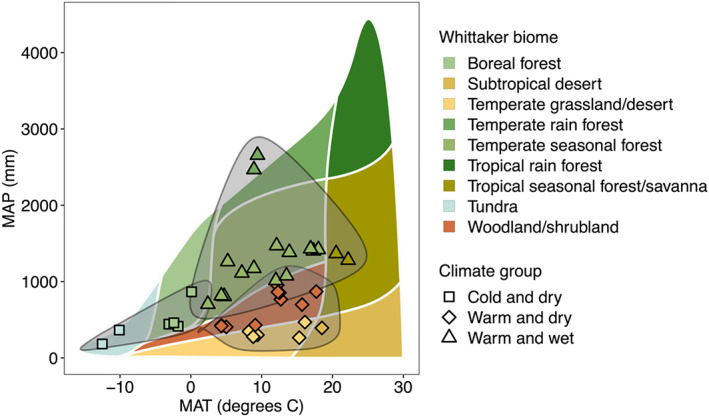
Determination of site climate groups. Sites are plotted according to the Whittaker biome temperature‐by‐precipitation framework (*plotbiomes* R package) (Ricklefs, 2008; Stefan and Levin, 2023; Whittaker, 1962). Points represent individual sites in this data set, which were assigned Whittaker biomes in prior work (Heckman et al., 2021; Nave, Heckman, et al., 2021). In this study, sites were further grouped into “cold and dry” (tundra and boreal forest), “warm and wet” (temperate rain forest, temperate seasonal forest, tropical seasonal forest/savanna), and “warm and dry” (woodland/shrubland, temperate grassland/desert, and subtropical desert) climate groups. Black lines on plot are polynomial spline curves derived from the “geom_encircle” function in *ggalt* (Rudis et al., 2017) and are provided for visualization of climate group divisions only.

Using National Ecological Observatory Network (NEON) soil temperature sensor data collected during 2018–2020 from 40 sites across 8 biomes in the United States (Figure 1), we tested seasonal predictors of freeze‐thaw cycle occurrence, frequency, and magnitude. We assessed how vegetation type (captured by biome), season, climate regime (mean annual temperature, MAT, difference between maximum and minimum air temperatures, and mean annual precipitation (MAP), and surface insulative properties (captured by precipitation as snow, PAS, and organic mat thickness) are related to FTC occurrence and frequency across the study sites. We hypothesized that the relative importance of climate and landscape factors in prediction of FTC occurrence and frequency would differ across biomes, due to the interactive nature of controls on soil temperature. Specifically, we hypothesized that relationships between mean annual temperature (MAT) and FTC would depend on climate group, which integrates ecosystem‐specific variation in insulative controls on soil temperature, such as vegetation type, PAS (as a proxy for extent of a persistent snowpack), and organic layer thickness. Understanding how the ecosystem controls on FTC vary across biomes is critical for the prediction of future FTC change under climate and environmental change, and is enabled by the broad spatial scale, diversity of ecosystem types, and auxiliary data available across the NEON network.

## 2 Materials and Methods

### 2.1 NEON Study Sites

**Figure 2:**
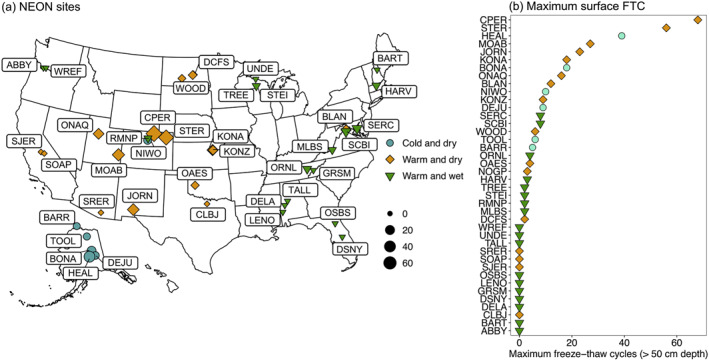
(a) Map of National Ecological Network (NEON) sites used for freeze‐thaw cycle quantification. The size of the points corresponds to the maximum number of Freeze‐thaw cycles (FTC) in the top 50 cm of the soil profile within a season across measurement years (09‐01‐2018 to 05‐30‐2020). (b) Ranking of maximum FTC within a season by site. For both (a, b), color and shape of the points corresponds to the assigned climate group, as described in Section 2.4. Full site names and site information are included in Table S1 and S2 in Supporting Information S1.

We used soil temperature data from 40 terrestrial NEON sites (Figures 1 and 2), including temperate grassland/desert, woodland/shrubland, boreal forest, temperate seasonal forest, tundra, subtropical desert, temperate rainforest, and tropical seasonal forest/savanna. These sites were selected based on availability of site and soil characterization data available in Nave, Heckman, et al. (2021), and complementarity to SOC cycling studies (Heckman et al., 2021; Nave, Bowman, et al., 2021; Possinger et al., 2021; Weiglein et al., 2022).

### 2.2 Software and Packages

All data processing and statistical analyses were conducted using R v. 4.2.3 (R Core Team, 2023) in RStudio 2023.03.1 + 446 (Posit Team, 2023), unless otherwise noted. We used the base R stats package (R Core Team, 2023) unless otherwise noted. We used the *ggplot2* (Wickham, 2016), *ggforce* (Pedersen, 2022), *ggalt* (Rudis et al., 2017), *usmap* (Di Lorenzo, 2023), and *soilpalettes* v0.1.0 (Patel & Bond‐Lamberty, 2022) packages for data visualization and mapping.

### 2.3 Data Acquisition and Processing

#### 2.3.1 Soil Temperature Sensor Data

Details of NEON soil temperature sensor installation, monitoring, and QA/QC can be found at the NEON data access portal (NEON, 2023). Soil temperature sensors (Thermometrics Climate PRT Probe, Thermometrics Corporation, Northridge, CA) at NEON sites are installed as part of a soil sensor array extending in a linear transect aligned with the dominant airshed at each site's eddy covariance flux tower (*n* = 5 sensor locations per site). In prior work (Rooney, Bailey, Patel, Dragila, et al., 2022; Rooney, Bailey, Patel, Possinger, et al., 2022), we used NEON sensor measurements to assess FTC frequency in permafrost soil systems, effectively capturing both >0 and < 0°C soil temperature fluctuations. Sensors are spaced at a distance designed to allow for spatially independent measurements (maximum = 40 m), accounting for site‐specific terrain limitations. At each position, up to 9 sensors are installed at approximately 2, 6, 16, and 26 cm‐depth increments at the surface, and at variable depths in deeper subsoils (NEON, 2023). For this study, we accessed the 2021 release of available site soil temperature data (NEON, 2021) using the *neonUtilities* package for R v.4.0.1 (Lunch et al., 2023) for the date ranges: 09‐01‐2018 to 11‐30‐2018 (fall 1), 12‐01‐2018 to 02‐28‐2019 (winter 1), 03‐01‐2019 to 05‐30‐2019 (spring 1), 06‐01‐2019 to 08‐31‐2019 (summer 1), 09‐01‐2019 to 11‐30‐2019 (fall 2), 12‐01‐2019 to 02‐29‐2020 (winter 2), and 03‐01‐2020 to 05‐30‐2020 (spring 2).

#### 2.3.2 Site Climate and Soil Data

Site names, locations, MAT, MAP, and MAP‐Hargreaves reference evaporation (Eref) were accessed from Nave, Heckman, et al. (2021). Site‐level PAS, annual maximum temperature, and annual minimum temperature were determined using ClimateNA v5.10 (https://tinyurl.com/ClimateNA, Wang et al., 2016). All climate variables represent 1961–1990 climate normals. Annual temperature difference (Tdiff) was calculated as the difference between mean warmest month temperature and mean coldest month temperature. Organic mat thickness was determined by calculating the total thickness of all organic (i.e., “O” horizon designation) horizons using profile descriptions from soil cores collected at each NEON site, as described in Nave, Bowman, et al. (2021).

### 2.4 Site Grouping Approach by Climate Regime

To increase evenness of site observations for statistical comparisons of climate and site effects on FTC across ecosystems, we grouped NEON sites into three broad “climate groups” based on Whittaker biome categorization (Figure 1). In prior work (Heckman et al., 2021; Nave, Bowman, et al., 2021), NEON sites were assigned Whittaker biomes based on plotted position on the MAT‐by‐MAP Whittaker biome framework (visualized using the *plotbiomes* R package) (Figure 1) (Ricklefs, 2008; Stefan & Levin, 2023; Whittaker, 1962). In this study, we grouped sites within adjacent Whittaker biome polygons into larger climate groups, where “cold and dry” sites (*n* = 6) correspond to boreal forest and tundra (MAT ‐12.5 to 0.1°C, MAP 183–866 mm); “warm and wet” sites (*n* = 18) correspond to temperate rain forest, temperate seasonal forest, and tropical seasonal forest/savanna (MAT 2.4 to 22.4°C, MAP 704–2,657 mm); and “warm and dry” sites (*n* = 16) correspond to subtropical desert, woodland/shrubland, and temperate grassland/desert (MAT 4.3 to 18.5°C, MAP 266–954 mm). Distributions of site and soil variables across climate groupings are shown in Figure S2 in Supporting Information S1.

### 2.5 FTC Quantification

We quantified FTC within each season using the *FTCQuant* R package (Bajcz, 2023). The *FTCQuant* freeze‐thaw quantification algorithm counts the number of times soil temperature rises above and below a defined threshold, for a defined period of time. Our FTC quantification approach was modified from the approach described in Rooney, Bailey, Patel, Dragila, et al. (2022) and Rooney, Bailey, Patel, Possinger, et al. (2022). We applied stringent data quality screening in order to account for variable continuity of soil temperature measurements across the 40‐site data set. Data screening code and annotated plots of all sensor measurements are available at: https://zenodo.org/doi/10.5281/zenodo.10654856).

Because the functionality of the FTC quantification algorithm (Bajcz, 2023) is sensitive to missing data, we excluded any individual sensor measurements (for a given season, depth, and replicate plot) with >3% missing data, or missing data at the start of the measurement period (Rooney, Bailey, Patel, Dragila, et al., 2022; Rooney, Bailey, Patel, Possinger, et al., 2022). In addition to directly screening sensors with missing data, we visually inspected individual sensor plots for each site by depth by season combination. While we confirmed that the majority of sensor measurements with poor continuity were excluded by filtering based on missing data, we manually excluded a small number (17) of sensor measurements with major deviations from replicate sensors that could potentially affect FTC counts. Sensors were manually excluded when signs of sensor failure were detected (e.g., flat‐line sensor readings, jumps from very high to very low temperature; see annotated plots at https://zenodo.org/doi/10.5281/zenodo.10654856). Because of the filtering of individual sensor measurements, some interpretations were limited to 1 year of data for a given combination of season, site, depth, and replicate sensor. A list of all excluded sensors is available as Supplementary Data (Data S1 in Supporting Information S1).

In addition to modifications to sensor data screening, we modified the FTC quantification approach in Rooney, Bailey, Patel, Dragila, et al. (2022) and Rooney, Bailey, Patel, Possinger, et al. (2022) to capture both rapid, lower‐magnitude FTC and longer, higher‐magnitude FTC, using two sets of FTC quantification parameters: (a) Temperature fluctuations above and below 0°C (±1.5°C) that persist for 4 hr or more (“4‐hr FTC”), (b) temperature fluctuations above and below 0°C (±2.0°C) that persist for 12 hr or more (“12‐hr FTC”).

### 2.6 Statistical Analysis

#### 2.6.1 Climate and Site‐Level Relationships With FTC Across Biomes

We tested two metrics of FTC as a function of continuous climate or site‐level property variables: presence/absence of freeze‐thaw (binary response; all 40 sites), or the maximum number of freeze thaw when FTC were present (continuous; sites with zero FTC excluded). We used maximum FTC because (a) it represents the greatest range in FTC across sites, (b) captures an upper limit of FTC that is expected to broadly increase over time (e.g., as shown in Figure S1 in Supporting Information S1). First, to test for relationships between climate/site variables and the overall presence or absence of FTC, we used a binomial (presence/absence) generalized linear model. In the binomial model, we evaluated the extent to which climate/site variables, climate group, and the interaction between them influenced the likelihood of FTC presence/absence (in any season over the measurement period) using a likelihood ratio chi‐square test and used the binomial model to predict likelihood of FTC occurrence across levels of each climate/site variable.

Second, we investigated factors influencing freeze‐thaw within sites where temperature fluctuations were large enough to generate freeze‐thaw and excluded sites with no freeze‐thaw (which were captured in our binomial model) (Figure S4 in Supporting Information S1). For this analysis, we used multiple linear regression model *F*‐tests to test the main effects of climate/site variable, climate group, and the interaction between each variable and climate group on the maximum number of FTC within a given season (i.e., winter, spring, summer, or fall FTC). By restricting this analysis only to sites and seasons with FTC present, some combinations of season by climate group resulted in <3 FTC and were excluded from MLR models. Within each season by climate group combination, we used Spearman rank correlation with continuity correction for estimated rho values to visualize the direction and magnitude of the relationship between climate/site variables and FTC.

## 3 Results

### 3.1 Patterns in FTC Duration and Occurrence Across Depth

**Figure 3:**
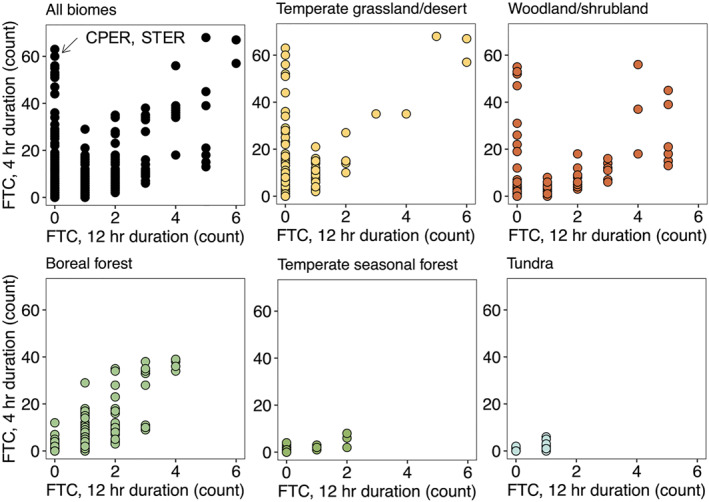
Relationship between rapid, lower‐magnitude temperature fluctuations (4‐hr freeze‐thaw cycles (FTC)) and longer‐duration, higher‐magnitude temperature fluctuations (12‐hr FTC) across all soil depths and sites, separated by biome. FTC count is the total number of FTC detected with a given season over the measurement period 09‐01‐2018 to 05‐30‐2020. The occurrence of rapid FTC without longer‐duration FTC is highlighted for two sites: Central Plains Experimental Range (Weld County, CO, USA) and North Sterling (STER) (Logan County, CO, USA).

Overall, FTC counts were strongly zero‐weighted for both rapid (4‐hr) and longer‐duration (12‐hr) parameters, partially driven by the nearly complete absence of FTC deeper than 0.5 m in the soil profile (Figure 3, Figures S3 and S4 in Supporting Information S1). However, we detected over 60 rapid FTC within a season across the 2‐year measurement period, the majority of which occurred within 0.5 m of the soil surface (Figure S3 in Supporting Information S1). Although 4‐hr and 12‐hr FTC were strongly correlated overall (Spearman's rho = 0.68, *p* < 0.001), some temperate grassland/desert and woodland/shrubland biomes experienced a large number (up to ∼60) rapid, low‐magnitude FTC that were not paired to longer‐duration FTC (Figure 3).

### 3.2 Patterns in Surface (0–50 cm) FTC Across Biomes and Climate Groups

**Figure 4:**
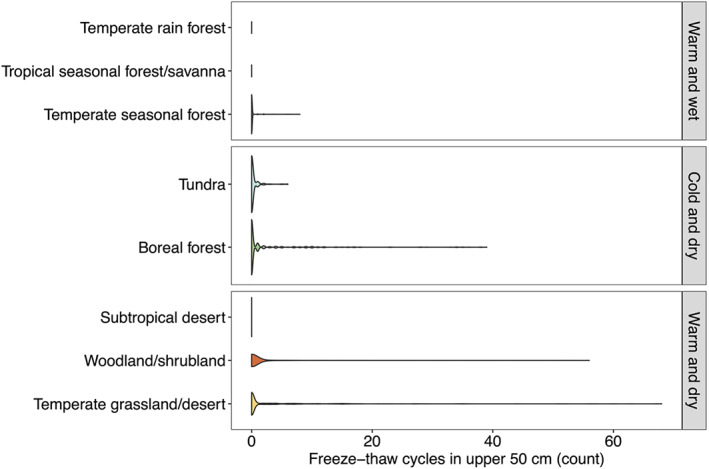
Distribution of 4‐hr freeze‐thaw cycles across all seasons and measurement years. Violin plots show mirrored density (Hintze & Nelson, 1998).

**Figure 5:**
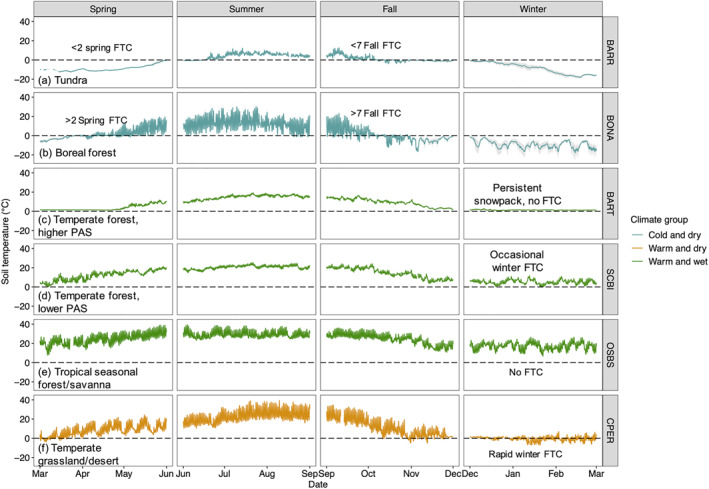
Surface (∼6 cm depth) temperature profiles for selected sites within cold and dry, warm and wet, and warm and dry climate groups. Lines are the average of sensors (1–5 sensors per depth per site), and shaded regions show standard deviation. Full site names, locations, and climate information are included in Table S1 and S2 in Supporting Information S1. FTC, freeze‐thaw cycle; PAS, precipitation as snow.

**Figure 6:**
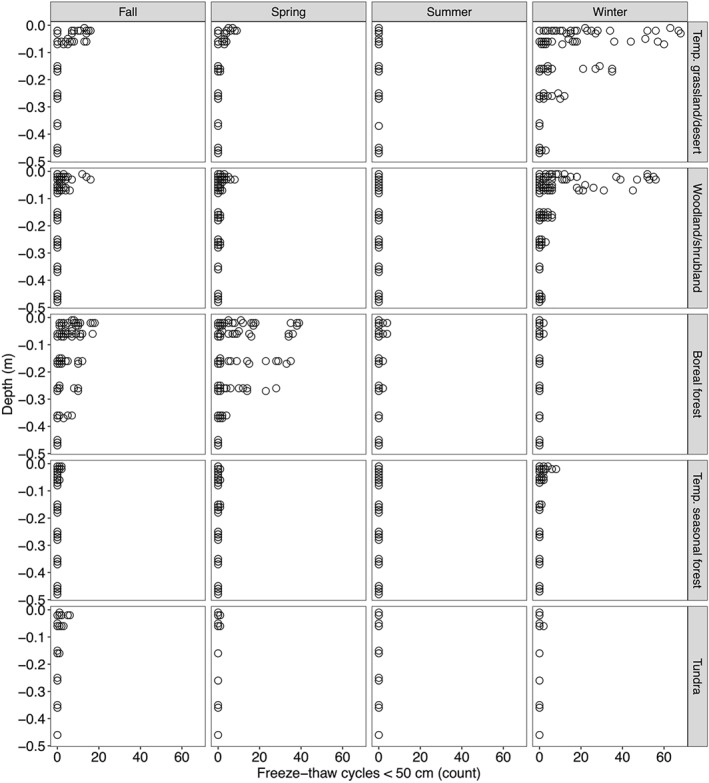
Depth profiles of freeze‐thaw cycles (FTC) in the upper 50 cm of the soil profile, separated by biome and season. All FTC within a season are shown across all available measurement years, across the period 09‐01‐2018 to 05‐30‐2020.

Because the majority of the FTC occurred in 0–50 cm depths, and more variation was detected for rapid, low‐magnitude (4‐hr) FTC (Figure S3 in Supporting Information S1), we further considered patterns in rapid surface FTC only across the NEON sites and biomes. The distribution of FTC remained zero‐weighted, but when FTC were detected, the maximum number of rapid surface FTC within a season ranged from 1 to over 60 FTC (Figure 4). No surface FTC were detected in subtropical desert, temperate rainforest, and tropical seasonal forest/savanna biomes, consistent with warm climates lacking persistent freezing soil and air temperatures (Figures 4 and 5e). In tundra sites, either 0 or few (up to 6) surface FTC were detected, consistent with relatively persistent frozen conditions (Figures 4 and 5e). However, a wide range of FTC (from 1 to over 60) were detected across temperate seasonal forest, boreal forest, woodland/shrubland, and temperate grassland/desert sites (Figures 4 and 6).

Overall, the highest number of FTC were in warm and dry sites, including woodland/shrubland and temperate grassland/desert biomes (Figures 4 and 6). In warm and dry sites, FTC mainly occurred in winter, and within the top 30 cm of the soil profile (Figures 2 and 5f, Figures S5, and S6 in Supporting Information S1). Fall and spring FTC were also present in warm and dry sites (Figure S5 in Supporting Information S1). Sites with the highest number of winter FTC also experienced the highest number of fall and spring FTC (up to 15 and 10 FTC in the fall and spring, respectively), which contributed to the highest total FTC observed across the 40‐site network in these sites (CPER and STER) (Figure 3, Figure S5 in Supporting Information S1). Consistent with the occurrence of rapid (4‐hr) FTC that were not correlated to longer (12‐hr) FTC (Figure 3), diurnal temperature fluctuations above and below 0 degrees C (±1.5°C) occurred regularly within the top 10 cm of the soil profile in temperate grassland/desert biomes (Figure 5f, Figure S7 in Supporting Information S1). Within the cold and dry climate group, the majority of the FTC occurred in boreal forest biomes (Figure 4). In contrast to warm and dry climates, boreal forest FTC largely occurred in fall and spring, and were more evenly distributed across the top 50 cm of the soil profile (Figures 2 and 5b, Figures S5 and S6 in Supporting Information S1).

#### 3.2.1 Relationships Between Site‐Level Climate and Soil Variables and FTC Presence/Absence

##### 3.2.1.1 Air Temperature

**Figure 7:**
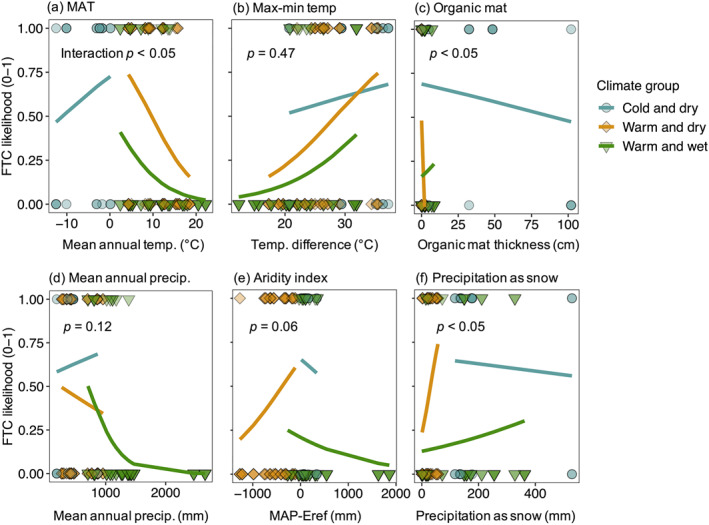
Relationships between climate and site variables and the presence or absence (binomial likelihood model) of freeze‐thaw cycles (FTC). Point shapes and point and line colors indicate climate groupings. On each plot, *p*‐values indicate the interaction term for climate groupings (i.e., whether the relationship between variables and FTC likelihood differs among climate groupings). Binomial model main effects for each variable and climate group are included in Table 1. (a) MAT = mean annual temperature; (b) Difference between annual maximum and minimum temperature; (c) Organic mat thickness; (d) MAP = mean annual precipitation; (e) Aridity index, or mean annual precipitation ‐ Hargreaves reference evaporation (Eref); and (f) Precipitation as snow.

Overall, higher MAT was related to reduced likelihood of FTC presence, but in cold and dry climates, higher MAT was tied to greater likelihood of FTC (interaction *p* < 0.05) (Figure 7a, Table 1). A greater difference between maximum and minimum annual temperatures, however, was strongly related to higher presence of FTC across all climate groups (Figures 7b and Table 1).

**Table 1:** Binomial Model of Site/Soil Variable Effects on the Likelihood of Freeze‐Thaw Cycle Occurrence (Presence/Absence)

Variable	Model term	DF	Deviance	Residual DF	Residual deviance	P(Chi)
Tdiff	Residual	NA	NA	150	195.71	NA
	Tdiff	1	25.9	149	169.81	0.000***
	Group	2	5.8	147	163.99	0.055*
	Interaction	2	1.5	145	162.49	0.471
MAP‐Eref	Residual	NA	NA	150	195.71	NA
	MAP‐Eref	1	2.0	149	193.7	0.156
	Group	2	16.9	147	176.78	0.000***
	Interaction	2	5.4	145	171.4	0.068*
MAP	Residual	NA	NA	150	195.71	NA
	MAP	1	24.7	149	171.03	0.000***
	Group	2	1.8	147	169.23	0.406
	Interaction	2	4.2	145	164.98	0.119
MAT	Residual	NA	NA	150	195.71	NA
	MAT	1	17.2	149	178.51	0.000***
	Group	2	12.1	147	166.44	0.002***
	Interaction	2	6.8	145	159.6	0.033**
Organic mat	Residual	NA	NA	150	195.71	NA
	Organic mat	1	1.1	149	194.63	0.298
	Group	2	18.4	147	176.22	0.000***
	Interaction	2	7.5	145	168.73	0.024**
PAS	Residual	NA	NA	150	195.71	NA
	PAS	1	1.0	149	194.7	0.314
	Group	2	18.9	147	175.81	0.000***
	Interaction	2	7.6	145	168.22	0.023**

*Note.* DF = degrees of freedom.

##### 3.2.1.2 Moisture Regime

Similar to MAT, higher MAP was related to lower likelihood of FTC presence in warm and dry and warm and wet climate groups, but increasing MAP had a slight positive effect on FTC in cold and dry climates (interaction *p* = 0.12) (Figures 7d and Table 1). In contrast, relationships between site‐level aridity (MAP—potential evapotranspiration, Eref) and FTC were inconsistent across climate groups (interaction *p* = 0.06). In warm and dry climates, higher aridity tended to increase the presence of FTC, while in warm and wet climates, higher aridity tended to decrease the likelihood of FTC presence (Figures 7e and Table 1).

##### 3.2.1.3 Precipitation as Snow

While PAS was not significantly related to the presence of FTC overall (main effect *p* = 0.31, Table 1) there was a significant interaction (*p* < 0.05) between PAS and climate group, indicating an environment‐specific relationship between PAS and the presence of FTC (Figures 7f and Table 1). In cold and dry climates, increasing PAS was associated with lower likelihood of FTC presence, whereas the inverse was observed for warm and dry and warm and wet climate groups.

##### 3.2.1.4 Organic Mat Thickness

Similar to PAS, organic mat thickness was not a significant overall predictor of FTC presence or absence (main effect *p* = 0.30), but the direction of this effect depended on climate group (interaction *p* < 0.05) (Figures 7c and Table 1). In cold and dry systems, greater organic mat thickness tended to decrease the likelihood of FTC. In warm and dry climates, higher organic mat thickness decreased the likelihood of FTC, while in warm and wet climates, the effect was weakly positive.

#### 3.2.2 Relationships Between Site‐Level Climate and Soil Variables and Number of FTC

##### 3.2.2.1 Air Temperature

**Figure 8:**
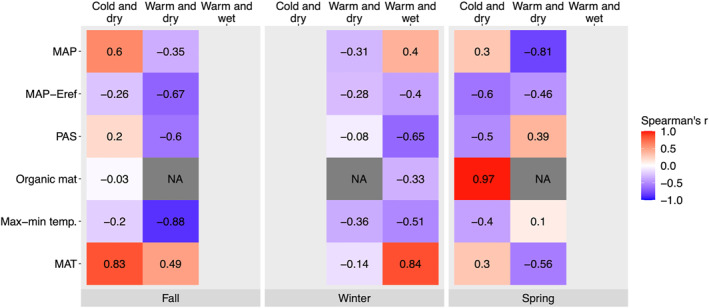
Direction and magnitude of relationships between climate and site variables and the number of freeze‐thaw cycles (FTC) (maximum per season over measurement record, 09‐01‐2018 to 05‐30‐2020). Values correspond to Spearman Rank correlation coefficient (r). MAP, mean annual precipitation; MAP‐Eref = MAP—Hargreaves reference evaporation; PAS, precipitation as snow; Organic mat = organic mat thickness; Max‐min temp. = annual maximum ‐ minimum temperature; MAT, mean annual temperature. Note that columns with no data were excluded due to too few FTC for analysis with the climate group and season combination.

Mean annual temperature was not related to the maximum number of fall FTC in any climate group. For spring FTC, MAT effects were not significant overall, but the direction of MAT effects differed between climate groups, with a slight positive effect in cold and dry climates, and negative effect in warm and dry climates (Figure 8, Table S3 in Supporting Information S1; interaction *p* = 0.06). In winter, increasing MAT had an overall positive effect on FTC frequency (main effect *p* = 0.05), and the effect of increasing MAT did not differ between climate groups (interaction *p* = 0.32). However, this relationship was driven mainly by a positive relationship between MAT and FTC in warm and wet climates (e.g., temperate forests).

In contrast to MAT, in warm and dry climates, increasing site‐level annual temperature difference (annual maximum–minimum) was negatively related to the number of fall FTC, an effect which was not present in cold and dry climates (Figure 8, Table S3 in Supporting Information S1; interaction *p* < 0.005). A negative relationship between temperature difference and winter FTC was also detected in both warm and dry and warm and wet climate groups (Figure 8, Table S3 in Supporting Information S1; main effect *p* < 0.05). There was no relationship between temperature difference and spring FTC or climate group interaction (main effect *p* = 0.72, interaction *p* = 0.82).

##### 3.2.2.2 Moisture Regime

In fall, MAP was not consistently related to the number of FTC for any climate group (Figure 8, Table S3 in Supporting Information S1; main effect *p* = 0.84, interaction *p* = 0.34). In both winter and spring, there was a weak negative relationship between MAP and FTC overall (Figure 8, Table S3 in Supporting Information S1; main effect *p* = 0.054 for winter and *p* = 0.061 for spring), driven mainly by a negative relationship between MAP and FTC in warm and dry climates.

Similarly, site‐level aridity (MAP—Hargreaves references evaporation, Eref) was not related to fall and spring FTC in any climate group (Figure 8, Table S3 in Supporting Information S1; main effect *p* = 0.86 for fall and 0.71 for spring). However, in winter, higher humidity (higher MAP‐Eref) was correlated to lower FTC overall, with no effect of climate group (Figure 8, Table S3 in Supporting Information S1; main effect *p* < 0.05; interaction *p* = 0.77).

##### 3.2.2.3 Precipitation as Snow

In spring, no effects or interactions related to PAS were detected (Figure 8, Table S3 in Supporting Information S1; main effect *p* = 0.64, interaction *p* = 0.43). In fall, PAS was not a predictor of FTC overall, but a significant interaction between PAS and climate group was detected (Figure 8, Table S3 in Supporting Information S1; interaction *p* < 0.05): in warm and dry climates, higher PAS was negatively related to fall FTC, while in cold and dry climates, a weak positive effect was detected (Figure 8). Higher PAS negatively related to winter FTC overall (main effect *p* < 0.05) across all climate groups, but the relationship was most apparent in warm and wet climates (Figure 8).

##### 3.2.2.4 Organic Mat Thickness

After reducing the number of observations to only sites with FTC present within a combination of season and climate group, a relationship between FTC and organic mat thickness could only be tested for cold and dry climates in fall and spring, and warm and wet climates in winter, so interaction effects between organic mat and climate groups are not applicable (Figure 8). However, within spring FTC for cold and dry sites only, we identified an overall relationship between increasing organic mat thickness and increasing FTC (main effect *p* < 0.01, Spearman's *r* = 0.97). However, we note that strong correlation is based on only six observations (Figure S8 in Supporting Information S1).

## 4 Discussion

### 4.1 Freeze‐Thaw Is a Predominantly Surficial Disturbance Process

The concentration of freeze‐thaw occurrence at the soil surface (<30 cm) supports an emphasis on freeze‐thaw as a predominantly surficial disturbance process in future climate projections and soil transformations (Figure 2). Our findings align with prior FTC quantification work which found dampening of temperature fluctuations in the soil subsurface (Henry, 2007) and land surface models that have previously modeled freeze‐thaw in surface soils from heat and energy transfer simulations (Hu et al., 2006). The concentration of FTC in biologically and hydrologically active surface horizons highlights the importance of freeze‐thaw effects on microbial biomass and respiration (Feng et al., 2007; Freppaz et al., 2007) and soil physical structure and porosity (Ge et al., 2024; Ma et al., 2021; Rooney, Bailey, Patel, Dragila, et al., 2022; Zhang et al., 2021). The same factors that drive heat and energy transfer (e.g., surface insulative properties) and the predominance of freeze‐thaw at the soil surface also amplify its sensitivity to future changes in climate and landscape factors.

Although the majority of biomes experienced freeze‐thaw at only surface depths, we emphasize the potential outsized impact of infrequent freeze‐thaw deeper in the soil profile, specifically in cryosphere landscapes where permafrost thaw and cryogenic processes can drive FTC at deeper depths (Rooney, Bailey, Patel, Possinger, et al., 2022). We detected FTC in the 25–35 cm depth at HEAL, a boreal forest site with continuous permafrost, in contrast with little to no FTC deeper than 20 cm at DEJU and BONA, boreal forest sites with discontinuous or variable extent of permafrost (Jorgenson et al., 2008) (Figure S9 in Supporting Information S1). Studies in Arctic landscapes have identified physical and biogeochemical impacts from small frequencies (<7 FTC) of freeze‐thaw to newly thawed permafrost soils (Qi et al., 2006; Rooney, Bailey, Patel, Dragila, et al., 2022; Rooney, Bailey, Patel, Possinger, et al., 2022). Although the ability to extend observations across permafrost sites more broadly requires integration of additional sites and permafrost extent, these contrasts emphasize the need for additional exploration of how permafrost presence mediates FTC depth patterns, particularly for summer FTC (Figure S9 in Supporting Information S1).

### 4.2 Warm and Dry Sites Experienced a High Frequency of Rapid Winter FTC

**Figure 9:**
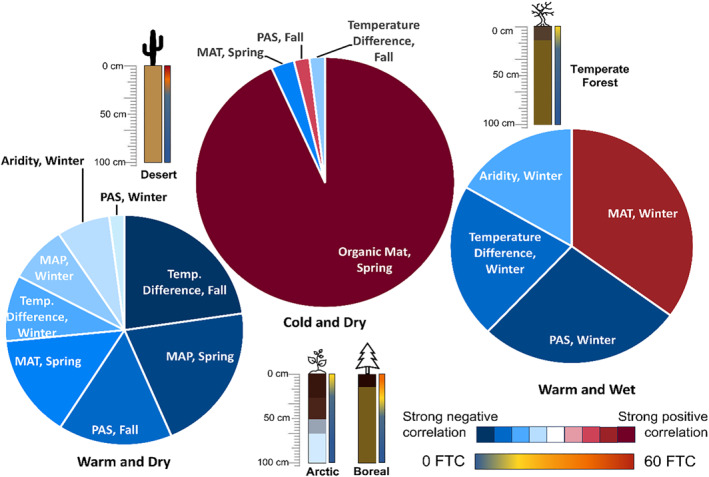
Summary figure of magnitude and direction of effect on seasonal freeze‐thaw cycle amount (significant effects only, based on MLR main effects). Pie chart wedge size and color corresponds to the Spearman rho coefficient values. All seasons are combined within the pie charts but are individually noted with each factor that has a significant effect. Specific biome examples and associated soil profiles and freeze‐thaw cycle frequency and depth are included.

Despite the consistency in FTC depth, there were large differences in the frequency of FTC across climate groupings (Figures 4, and 9). To our knowledge, the importance of rapid winter FTC in warm and dry soil systems relative to other sites and biomes as observed in our study (Figures 3 and 4) has not been reported by previous literature. Desert freeze‐thaw constitutes a significant gap in the cross‐biome freeze‐thaw literature, with the majority of reviews, field studies, and lab experiments focusing on tundra, alpine, temperate and boreal forests, grassland, hardwood, and agricultural soils (Henry, 2007; Qi et al., 2006; Song et al., 2017; Zhao et al., 2021). While underrepresented in the cross‐biome FTC literature, the potential for diurnal occurrences of freeze‐thaw and the importance of winter FTC in desert soils have been noted in the ability of FTC to increase winter soil respiration (Liu et al., 2016), alter nutrient cycling dynamics and associated enzyme activities under biocrusts (Zhang et al., 2023), and impact total carbon and nitrogen of biological soil crusts (Wang et al., 2015). Given these potential biogeochemical implications of rapid freeze‐thaw in arid systems, our findings motivate a need for application of advanced analytical tools (e.g., in Rooney, Bailey, Patel, Dragila, et al. (2022)) to probe rapid, diurnal freeze‐thaw effects on surface soil physical, chemical, and biological processes in warm and dry climates.

Despite the high number of overall FTC and notable winter patterns in warm and dry climates, the relationships we identified between site properties and either occurrence or frequency of FTC were complex, with no clear overarching site variable correlated to FTC in these systems. We posit that the general site‐level, annual average climate variables used may not capture controls on the diurnal or near‐diurnal temperature fluctuations at the soil surface that account for FTC in these systems. Therefore, future freeze‐thaw investigations in grassland and desert systems should assume the possibility of diurnal or near‐diurnal freeze‐thaw during winter, and together with parameterization of FTC quantification to capture rapid cycles (e.g., 4‐hr), interrogate system‐specific variables tied to shallow soil temperature fluctuations.

### 4.3 MAT and PAS Relationships Are a Key Determinant of Winter FTC

Mean annual air temperature acted as a primary control on FTC across all sites, in that the proximity of MAT to 0°C determined whether sites were too cold or too warm for temperature fluctuations (driven by other climate and landscape factors) to induce FTC (Figures 7 and 8). These findings suggest that the relationship between current MAT and the timing of FTC occurrence will be a key indicator of how FTC may change under future climate warming. For example, air temperatures at cold and dry sites (BARR and BONA) are currently too low in winter for FTC, but allow for spring and fall freeze‐thaw (Figure 5). In sites with relatively high MAT within the cold and dry climate group (e.g., BONA, Figure 5), winter temperature fluctuations were present, so rising winter air temperatures may generate conditions for potential future increases in winter FTC occurrence earlier in these systems than in colder cold and dry sites (e.g., BARR). This pattern is consistent with a prior, 31‐site study across Canada that found a negative relationship between rising air temperatures and soil freezing days, predicting a strong effect on FTC in winter (Henry, 2008). In contrast, warm and dry sites (e.g., CPER) experienced soil temperature fluctuations just above and below 0°C in winter, creating high frequencies of freeze‐thaw (Figure 5). As such, rising air temperatures could shift soil temperature fluctuations above a 0°C minimum, decreasing FTC frequencies in desert and grassland landscapes in coming decades.

While the insulating properties of snowpack and the reduction of temperature fluctuations is widely reported (Fu et al., 2017; Groffman et al., 2001a, 2001b; Hardy et al., 2001; Yi et al., 2019), our findings indicate that effects of MAT and PAS on FTC are not standardized within ecosystems. We identified unique outcomes from the relationship between MAT and PAS across climate gradients with diverging impacts to FTC indicating the potential for changing frequency in FTC under future rising air temperatures and changing winter conditions observed across climate gradients (Contosta et al., 2019; Hardy et al., 2001; Henry, 2007). In warm and wet sites, which are generally distributed across an eastern US latitudinal gradient (Figure 6), both higher PAS and lower air temperatures increased the likelihood of FTC occurrence (Figure 7; Figure S10 in Supporting Information S1). These observations align with findings from the Songnen Plain hinterland (a temperate continental monsoon region) in that the depth and timing of snowpack drove variations in soil freezing and thawing throughout the fall and winter (Fu et al., 2017). The occurrence of PAS (i.e., PAS > 0 mm) began at annual MAT < 15°C, the cutoff below which all FTC were also detected (Figure S10 in Supporting Information S1). However, with MAT < 15°C, more persistent snowpack (e.g., BART) stabilized soil temperatures, reducing fluctuations and the number of FTC (Figures 5, 8 and 9), consistent with studies of snowpack and soil temperature dynamics in other northern US forests (Wilson et al., 2020). In comparison, warm and wet sites with much lower PAS (e.g., SCBI) saw occasional winter FTC due to the lack of snowpack (Figure 5), likely driven by fluctuations in air temperature or instability in surface temperature under different types of ground cover (Li et al., 2024). When no PAS was present (e.g., OSBS), overall warm air temperatures did not allow for FTC in winter (or any other season) (Figure 5). These patterns suggest that with warmer air temperatures, mid‐latitude sites with current MAT of 10–15°C (e.g., SCBI, ORNL, GRSM, and SERC) will experience lower frequency of FTC, while sites with currently lower air temperatures and higher PAS (e.g., BART and RMNP) may experience higher FTC due to shorter duration or less continuous snowpack. However, changes in soil freezing because of changes to complex snowpack dynamics cannot be directly inferred (Wilson et al., 2020), motivating continued evaluation of complex winter climate dynamics modified by both vegetation cover and temporal variations in snowpack and air and soil temperatures (Contosta et al., 2019; Groffman et al., 2001a, 2001b).

In contrast to warm and wet sites, these MAT and PAS relationships with FTC were not found in the cold and dry group. Previous cryosphere studies have identified the role of snowpack to be modified by seasonality (Park et al., 2015; Rixen et al., 2022; Zhang, 2005). In winter and fall, snowpack in permafrost systems acts as an insulator from cold air temperatures, with snow cover increasing ground temperatures in the discontinuous permafrost region of Alaska (Goncharova et al., 2019; Osterkamp & Romanovsky, 1996) and triggering permafrost loss (Park et al., 2015). Prolonging soil thaw prior to freezing is consistent with our finding that increasing PAS decreases the probability of FTC in cold and dry climates (Figure 7). In contrast, spring snowpack may insulate against warming air temperatures, resulting in a delay in thaw similar to that caused by thick organic layers in boreal forest landscapes (Ahmed et al., 2021). Overall, the contrasting role of PAS in driving FTC in different climate groupings is a direct reflection of how MAT (encompassing incoming solar radiation and sensible heat flux) interacts with ground surface properties to mediate fluctuations in sensible heat transfer across systems (Figures 5, 6, 7, 8, 9).

### 4.4 Seasonality Drove Impacts of Organic Mat Thickness in Cold and Dry Sites

The contrasting effects of thicker organic layers at the soil surface (here referred to as organic mats) highlighted the complexity of soil surface properties as modifiers of belowground temperature dynamics. Increasing organic mat thickness decreased the likelihood of FTC occurrence (presence or absence) in cold and dry sites (Figure 7), aligning with previous findings that thick peat horizons at the soil surface are associated with the insulation of permafrost‐affected soils against changing shoulder season air temperatures and indicating that insulation hindered freeze‐thaw occurrence (Johnson et al., 2013; Shur & Jorgenson, 2007). Accumulation of peat layers at the soil surface has been associated with cooler soil temperatures, resulting in permafrost development and aggradation in cryosphere landscapes (Shur & Jorgenson, 2007). In contrast to overall likelihood of FTC occurrence, increasing organic mat thickness in cold and dry sites had a strong positive correlation with the number of FTC in spring (Figure 8). A possible interpretation is that thicker organic mats prolong freezing temperatures in the subsurface while air temperature warms, extending the spring thawing period and any freeze‐thaw that occurs within it. The role of organic mats in delaying spring thaw was reported in boreal forest landscapes of Western Canada where greater insulation of subsurface soils under thicker soil organic layers prolonged soil freeze during warming spring temperatures (Ahmed et al., 2021). Thermal conductivity of organic soils increases with higher moisture content (Hayashi, 2013), with further research needed to understand how spring snow melt and warming air temperatures influence surface FTC.

### 4.5 Vegetation and Microtopography May Modify FTC

Although our study identified differences in freeze‐thaw cycle frequency by depth, site, biome, and climate grouping, within‐site variability (created by vegetation, permafrost extent, and ground surface characteristics) remains an important area for future evaluation of freeze‐thaw cycle characteristics and drivers. Organic mat thickness was highly variable across cold and dry sites, ranging between 0 and 100+ cm (Figure S2 in Supporting Information S1). Sites with thick organic mats may contain strong within‐site variability, with hummocks, high and low center ice wedge polygons, and palsas showing inherent variation in organic layer thickness across the ground surface (Laberge & Payette, 1995; Mackay, 1980; Seppala et al., 1991). There is little previous work on how soil microtopography and cryogenic features influence small scale differences in freeze‐thaw cycle frequency, depth, and seasonality. A discontinuous permafrost study found differences in freeze‐thaw cycle frequency under open versus closed canopy cover along a forested hillslope, with warmer temperatures under open canopy resulting in less surface freeze‐thaw (∼5 cm) but greater subsurface freeze‐thaw (∼75 cm) compared with soils under closed canopy (Rooney et al., 2023). Microclimates and subsequent impacts to soil development (including organic layer thicknesses) and thermal regimes were influenced by slope aspect in catenas of similar topographies (Ping et al., 2005).

Processes driven by freeze‐thaw (including frost heave, ground subsidence, cryoturbation, and solifluction) may provide additional modifiers to freeze‐thaw distribution by altering both ground surface and subsurface characteristics (such as bulk density, organic matter content, or shifts in vegetation type and coverage), with impacts on thermal conductivity and sensible heat flux at the soil surface (Gruber, 2020; Mackay, 1980; Osterkamp & Romanovsky, 1996; Shur & Jorgenson, 2007). The occurrence of freeze‐thaw at both the top and bottom of the active layer as well as late summer freeze‐thaw processes have been observed in previous Arctic research from cryoturbation and hummock field observations in the Northwest Territories in Canada (Mackay, 1980). As Arctic landscapes continue to warm, it is notable that freeze‐thaw conditions may show unique characteristics (such as deeper depths and summer freeze‐thaw), requiring freeze‐thaw quantifications that allow for detection of these uncommon temperature fluctuations.

## 5 Conclusions

The potential for freeze‐thaw to act as a source of land and soil disturbance necessitates our ability to predict future occurrence and frequency. Our study highlights that direction and magnitude of correlations between drivers of freeze‐thaw (MAP, aridity, PAS, organic mat thickness, max‐min temperature, and MAT) and freeze‐thaw cycle amount differ by seasons and climate, information critical for predictions of general freeze‐thaw cycle trends using broad biome comparisons as climate and landscape properties undergo change. The likelihood of freeze‐thaw occurrence did not always indicate whether freeze‐thaw frequency would increase. For example, while greater organic mat thickness increased freeze‐thaw cycle frequency in spring for “cold and dry” sites, organic mat thickness increases decreased the overall likelihood of freeze‐thaw occurrence in the “cold and dry” sites across all seasons, demonstrating the complexity of freeze‐thaw drivers and how they interact with site‐specific microtopography and soil conditions. Predicting how a collection of sites within a biome may respond to shifts in dynamic climate factors with global warming is not the same as predicting how climate change will alter microsites and freeze‐thaw within a single site. Therefore, further evaluations of within‐site variability in freeze‐thaw driven by differences in vegetation structure and canopy is needed to capture landscape‐scale patterns. The buffering effects of vegetation and canopy can prolong soil freezing into spring months (Chen et al., 2020) and modify the impact of other dynamic site properties (Liston et al., 2007; Mott et al., 2018). In addition, interactions among soil moisture, soil temperature, and FTC may further mediate patterns of FTC, but were not assessed in this study due to the difficulty in attaining accurate soil moisture sensor data during periods of freezing. The quantification of FTC across sites, soil depth, and seasons in this study may enable future work that accounts for canopy structure, soil moisture, and intra‐site variability, improving predictive accuracy of future freeze‐thaw processes.

## Conflict of Interest

The authors declare that have no conflicts of interest to declare.

## Supporting information

Supporting Information S1

Table S1

Table S2

Table S3

## Data Availability

Data import, data processing, and statistical analysis code are available at: https://github.com/Erin‐Rooney/neon‐ftc‐b (https://10.5281/Zenodo.10654857; Rooney & Possinger, 2024). All raw soil temperature NEON data is cited in the manuscript, and available for download via NEON data portal (https://data.neonscience.org/data‐products/explore), or via download code in “code_for_rproj.” Supporting site and soil data are available through the Environmental Data Initiative (https://edirepository.org/) as cited in the manuscript. Additional site and soil data (organic mat thickness, precipitation as snow) is available as raw data at https://github.com/Erin‐Rooney/neon‐ftc‐b (https://10.5281/zenodo.10654857, available at https://zenodo.org/doi/10.5281/zenodo.10654856; Rooney & Possinger, 2024).
