# Supplementary material for: Climate and Ecosystem Factors Mediate Soil Freeze‐Thaw Cycles at the Continental Scale
Source: J Geophys Res Biogeosci. 2024 Nov 27;129(12):e2024JG008009. doi: 10.1029/2024JG008009 (PMC11600542; doi:10.1029/2024JG008009)
Supplement: Supplementary file 1 — Supporting Information S1 [file JGRG-129-0-s004.pdf]

Journal of Geophysical Research: Biogeochemistry

Supporting Information for

**Climate and ecosystem factors determine presence, frequency, and depth of soil freeze-thaw at the continental scale**

Erin C. Rooney[1,2] & Angela R. Possinger[3*]

1 National Soil Survey Center, USDA-NRCS, Lincoln, NE, USA

2 Earth and Planetary Sciences, University of Tennessee Knoxville, Knoxville, TN, USA

3 School of Plant and Environmental Sciences, Virginia Tech, Blacksburg, VA, USA

*Corresponding author: Angela Possinger (arp264@vt.edu)

## Contents of this file

Figures S1 to S10

## Additional Supporting Information (Files uploaded separately)

Captions for Tables S1 to S3

## Introduction

This supporting information file contains supplementary 10 supplementary figures for the accompanying manuscript "Climate and ecosystem factors determine presence, frequency, and depth of soil freeze-thaw at the continental scale."

**Supplementary Figures**

**Figure S1.** Measured changes in soil freeze-thaw cycles at the NRCS monitoring site in Toolik, Alaska across a 16-year period (data from USDA NRCS; Soil Survey Staff, 2020). These data illustrate the documented phenomenon of increasing FTC over the past 16 years of measurement records across at Toolik Field Station in Alaska. While freeze-thaw is increasing in this cold climate, other sites may see decreases in freeze-thaw cycle frequency where it was previously higher.

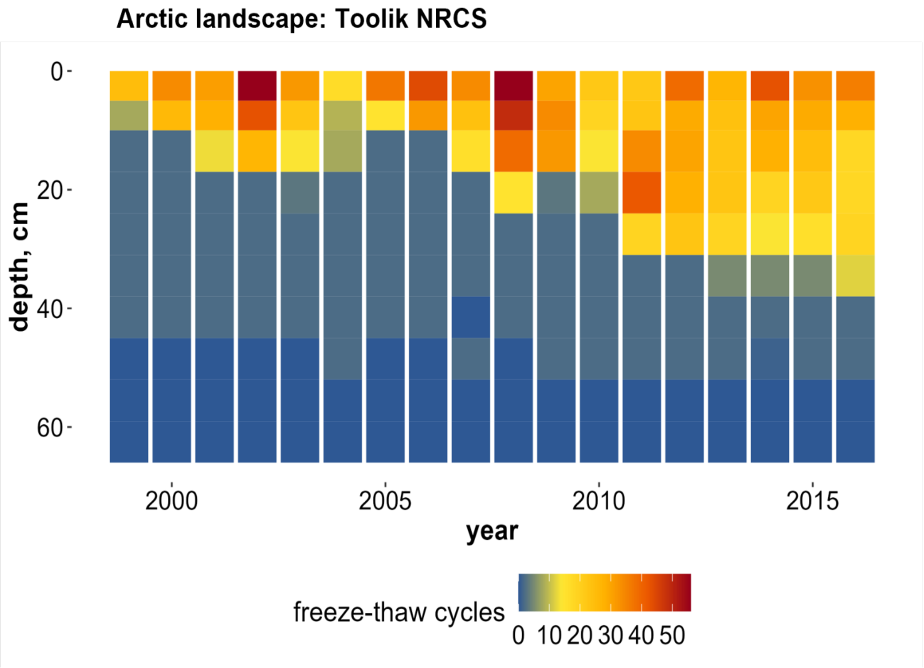

**Figure S2**. Distribution of climate variables and organic mat thickness across climate groups. Climate variables were derived are 1961-1990 climate normals derived from Nave et al. (2021a) and the ClimateNA database (Wang et al., 2016). (a) Mean annual temperature; (b) difference between annual maximum (mean warmest month temperature) and minimum (mean coldest month temperature); (c) organic mat thickness, based on organic horizon thickness; (d) mean annual precipitation (MAP); (e) MAP - Hargreaves reference evapotranspiration (Eref); (f) precipitation as snow (PAS).

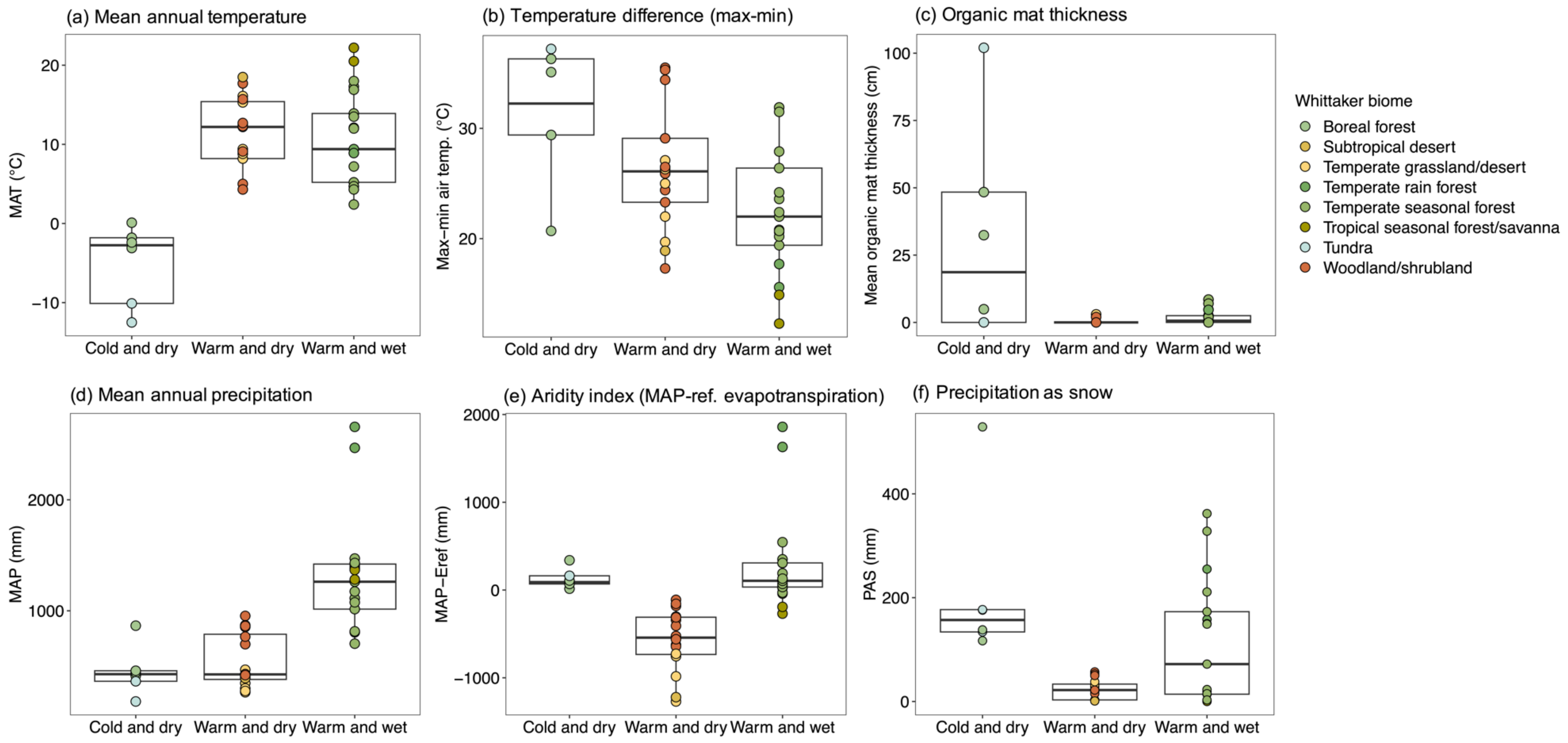

**Figure S3**. Total number of detected freeze-thaw cycles (across measurement period from 09-01-2018 to 05-30-2020 within a season) for rapid, lower-magnitude temperature fluctuations (4-hr FTC) and longer-duration, higher-magnitude temperature fluctuations (12-hr FTC).

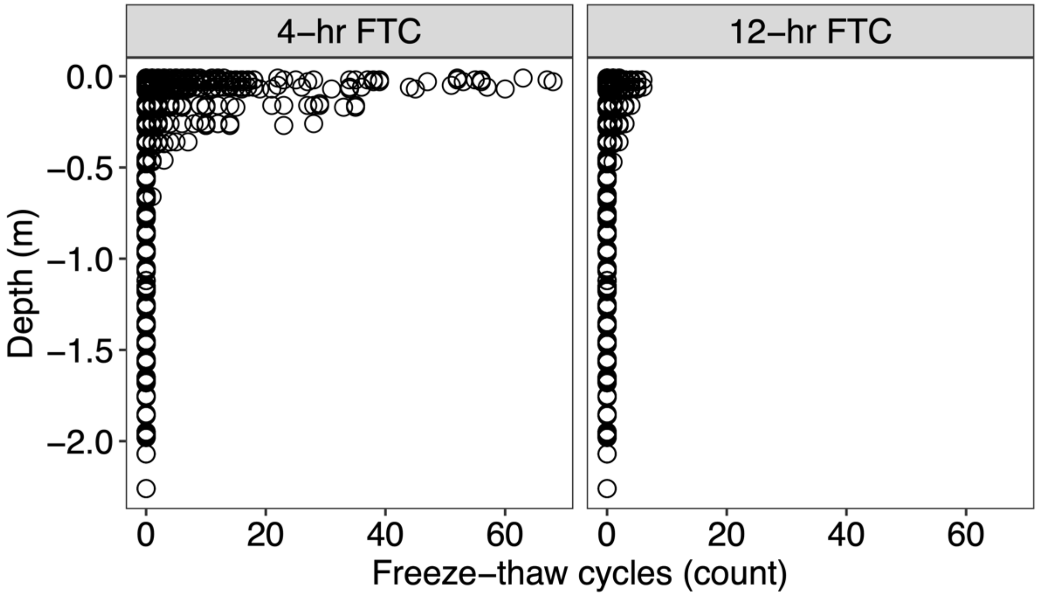

**Figure S4.** Distribution of total (measurement period 09-01-2018 to 05-30-2020) FTC counts within a season for rapid, lower-magnitude temperature fluctuations (4-hr FTC) (a) and longer-duration, higher-magnitude temperature fluctuations (12-hr FTC) (b) across all soil depths and sites. Inset plots show detail of count distributions greater than 0.

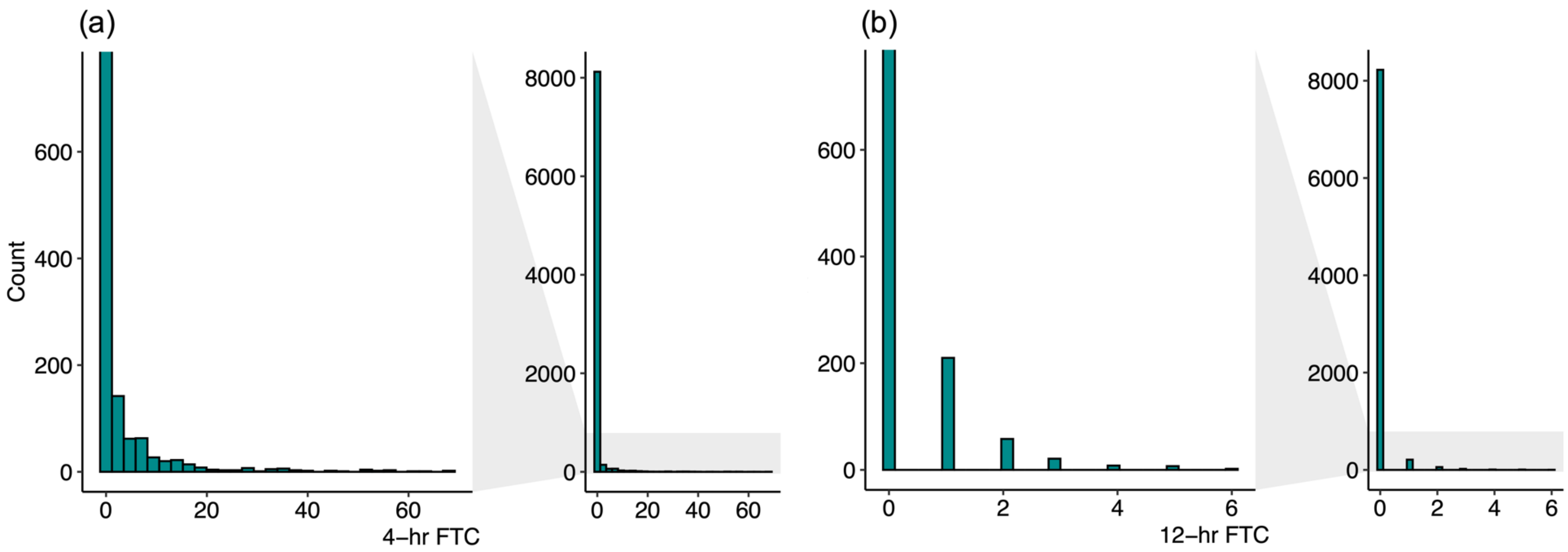

**Figure S5.** Distribution of 4-hr freeze-thaw cycles shown by season across all measurement years (09-01-2018 to 05-30-2020). Violin plots show mirrored density (Hintze & Nelson, 1998).

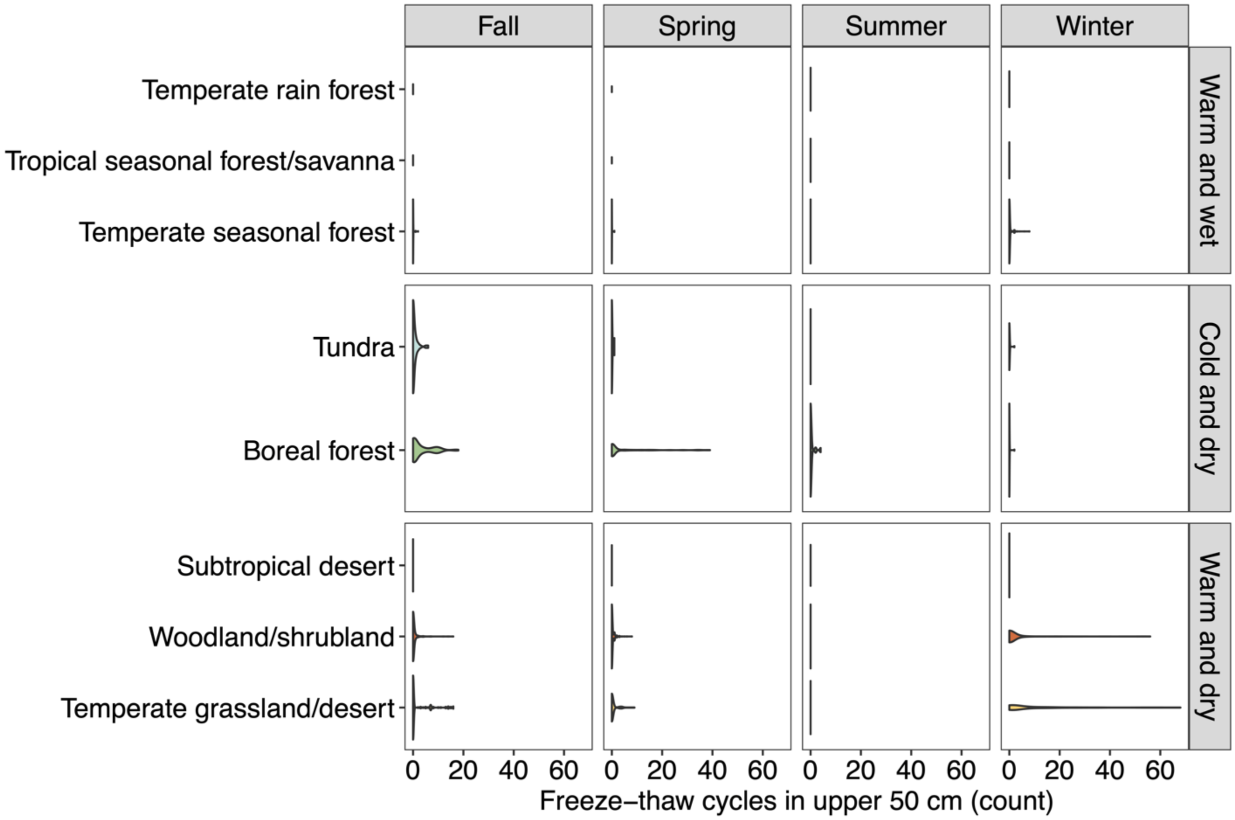

**Figure S6**. Ranking of maximum surface FTC within a season for each site over the measurement period of 09-01-2018 to 05-30-2020.

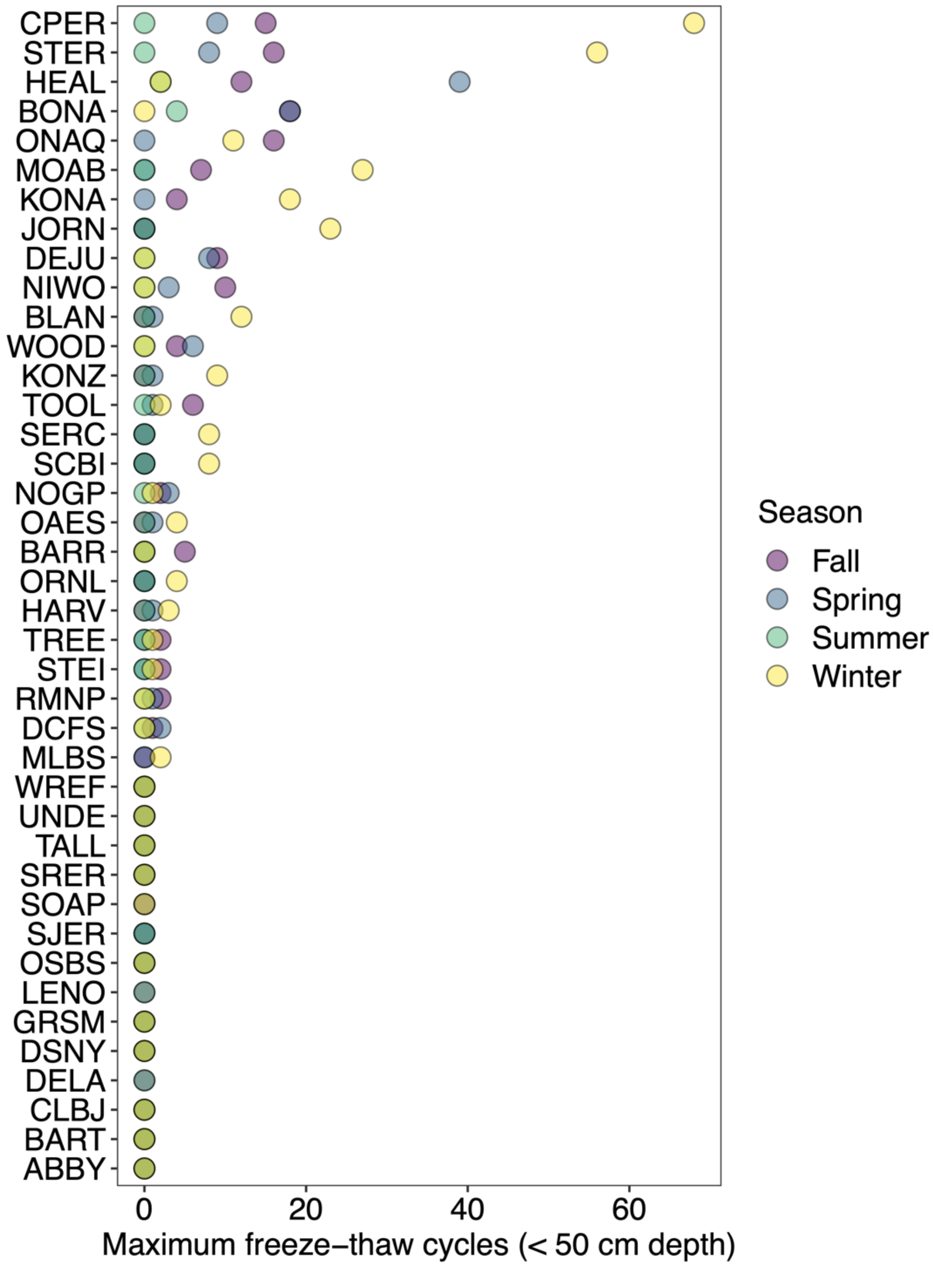

**Figure S7**. Detail of surface (~6 cm) winter soil temperature at the Central Plains Experimental Range (CPER) NEON site for a 10-day period (January 01-10 2020). Shaded area shows standard deviation of soil sensor locations (n = 5 sensors).

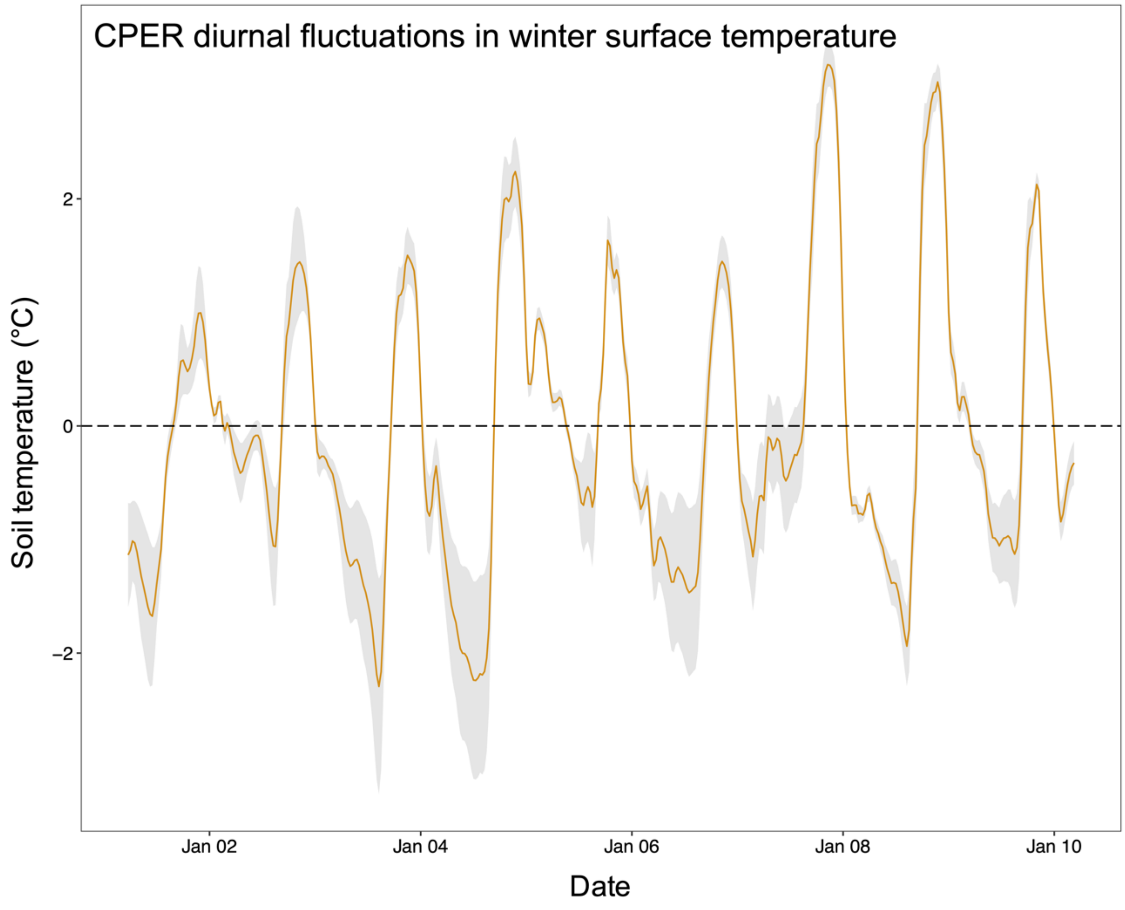

**Figure S8**. Correlation between organic mat thickness and site-level maximum FTC for spring FTC in cold and dry climates (tundra and boreal forest).

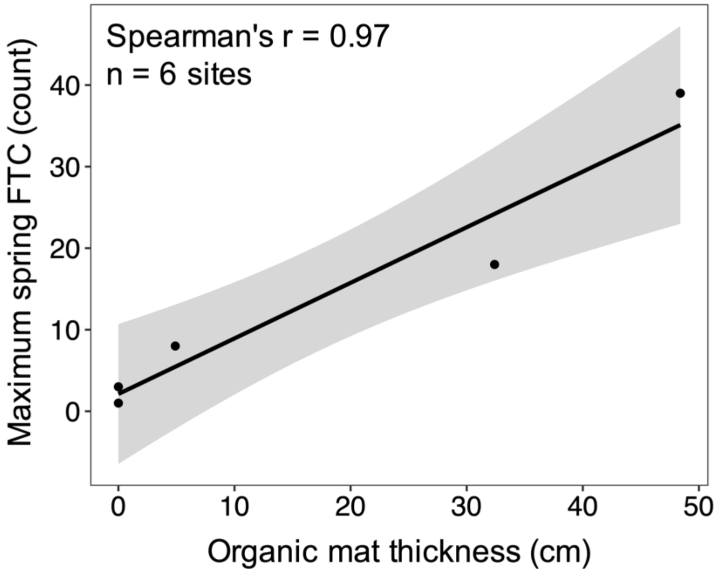

**Figure S9.** Surface (upper 50 cm) freeze-thaw cycles (FTC) for boreal forest sites only. Full site names and site information are defined in **Table S1** and **Table S2**.

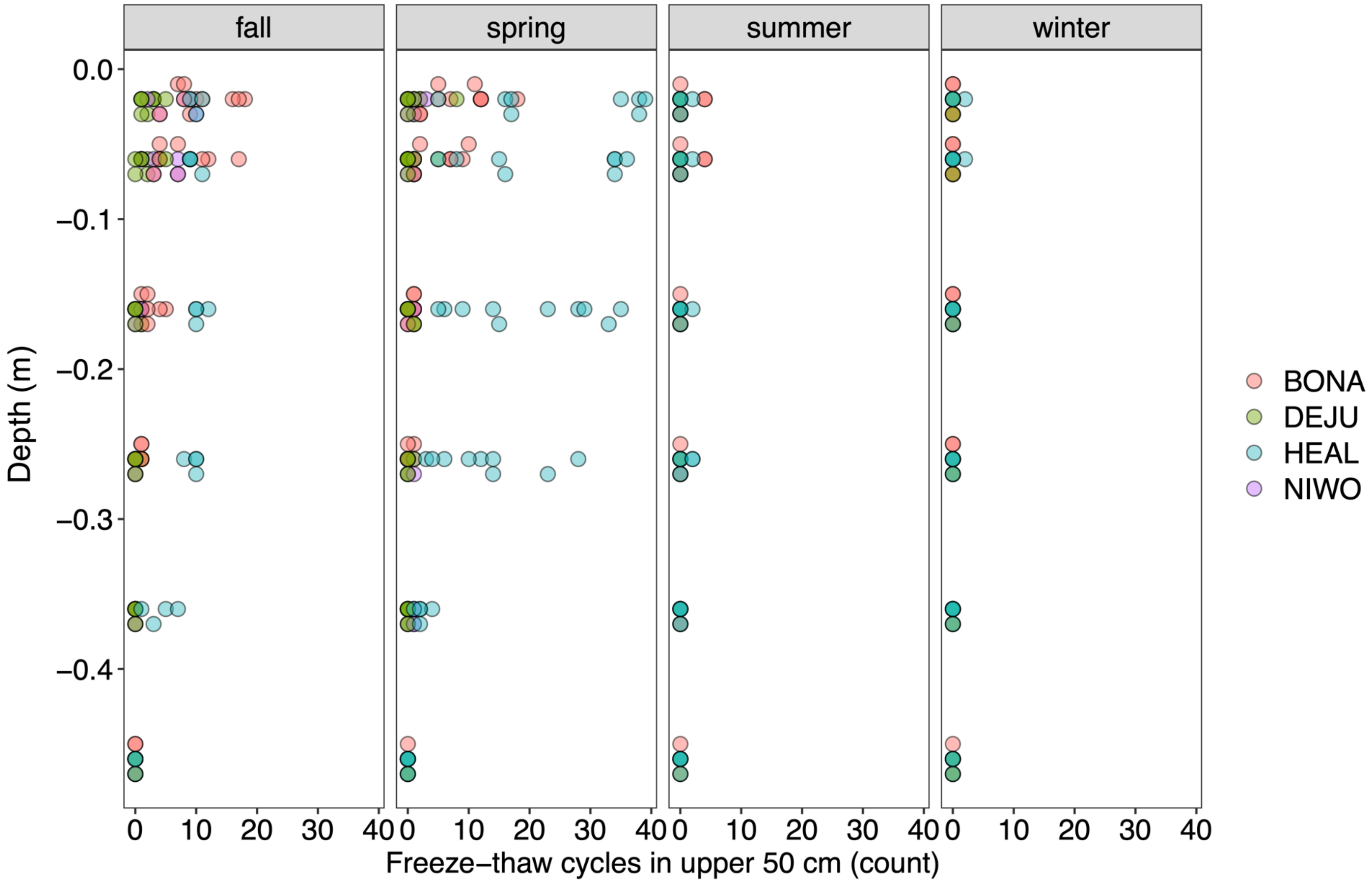

**Figure S10**. Relationship between mean annual temperature (MAT) and precipitation as snow and maximum freeze-thaw cycle (FTC) count (across all measurement years and seasons) for warm and wet climate sites.

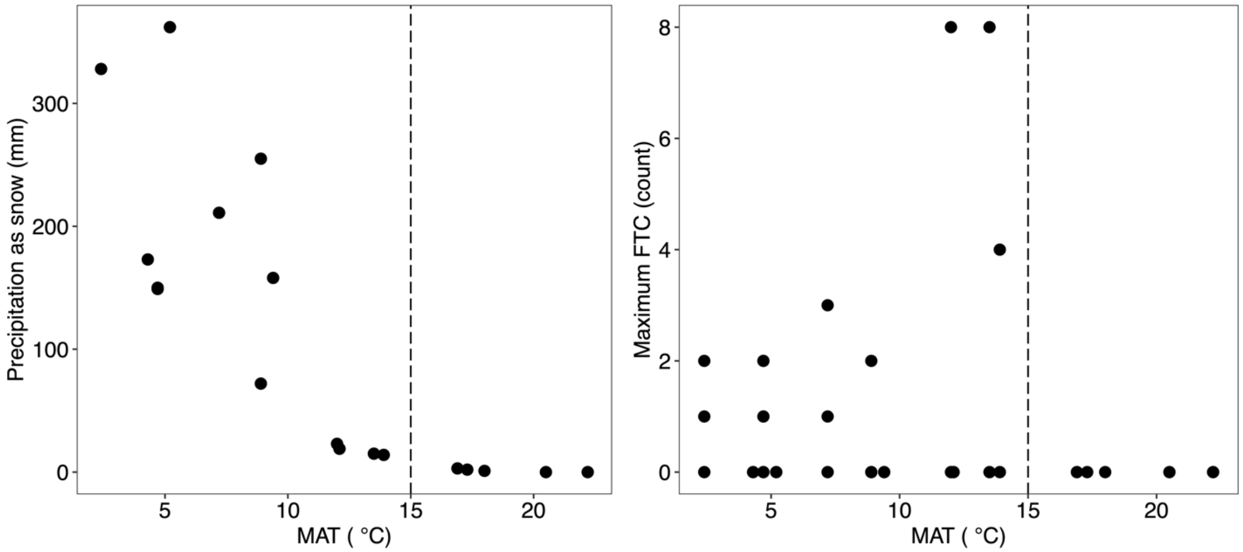

## Supplementary Tables

**Table S1**. Summary of site descriptive information and climate variables. MAT = Mean annual temperature; Tdiff = maximum – minimum annual air temperature; MAP = mean annual precipitation; MAP-Eref = MAP – Hargreaves reference evapotranspiration; PAS = precipitation as snow; WW = warm and wet; CD = cold and dry; WD = warm and dry; TRF = temperate rain forest; T = tundra; TSF = temperate seasonal forest; BF = boreal forest; WS = woodland/shrubland; TGD = temperate grassland/desert; TSFS = tropical seasonal forest/savanna; SD = subtropical desert. Full site names are included in **Table S2**.

**Table S2.** List of full site names.

**Table S3**. Multiple linear regression model F-test (ANOVA) results. For each site or soil variable, model terms included the main effects of each variable, climate group (warm and wet, warm and dry, cold and dry), and the interaction between these terms on the maximum number of freeze-thaw cycles (FTC) within a given season. DF = degrees of freedom; SSE = sum of squared error; MSE = mean squared error. Adjusted $R^2$ applies to the overall multiple linear regression model.

## Supplementary References

Hintze, J. L., & Nelson, R. D. (1998). Violin Plots: A Box Plot-Density Trace Synergism. *The American Statistician, 52*(2), 181–184. https://doi.org/10.1080/00031305.1998.10480559

Nave, L. E., Heckman, K. A., Bowman, M., Gallo, A. C., Hatten, J., Matosziuk, L., Possinger, A. R., SanClements, M., Strahm, B., Weiglein, T. L., & Swanston, C. (2021a). Soil Organic Matter Mechanisms of Stabilization (SOMMOS) - enhanced soil characterization data from 40 National Ecological Observatory Network (NEON) sites ver 2. *Environmental Data Initiative.* https://doi.org/10.6073/pasta/4d5f03a4619e834c031ab4a6a121de12 (Accessed 2023-08-11).

Soil Survey Staff, Natural Resources Conservation Service, United States Department of Agriculture. (2020). Soil Climate Research Station Data [Toolik, Alaska]. Available at: https://www.nrcs.usda.gov/resources/data-and-reports/soil-climate-research-stations

Wang, T., Hamann, A., Spittlehouse, D. L., & Carroll, C. (2016). Locally downscaled and spatially customizable climate data for historical and future periods for North America. *PLoS One*, *11*(0156720). https://doi.org/10.1371/journal.pone.0156720
