# Supplementary material for: Climate and Ecosystem Factors Mediate Soil Freeze‐Thaw Cycles at the Continental Scale
Source: J Geophys Res Biogeosci. 2024 Nov 27;129(12):e2024JG008009. doi: 10.1029/2024JG008009 (PMC11600542; doi:10.1029/2024JG008009)
Supplement: Supplementary file 2 — Table S1 [file JGRG-129-0-s001.pdf]

Supplementary Tables

Table S1. Summary of site descriptive information and climate variables. MAT = Mean annual temperature; Tdiff = maximum – minimum annual air temperature; MAP = mean annual precipitation; MAP-Eref = MAP – Hargreaves reference evapotranspiration; PAS = precipitation as snow; WW = warm and wet; CD = cold and dry; WD = warm and dry; TRF = temperate rain forest; T = tundra; TSF = temperate seasonal forest; BF = boreal forest; WS = woodland/shrubland; TGD = temperate grassland/desert; TSFS = tropical seasonal forest/savanna; SD = subtropical desert. Full site names are included in **Table S2**.

Site	Latitude	Longitude	Whittaker biome	Site climate group	MAT	Tdiff	MAP	MAP-Eref	PAS	Organic mat thickness
	Decimal °	Decimal °			°C	°C	mm	mm	mm	cm
ABBY	45.76243	-122.33033	TRF	Warm and wet	9.4	15.6	2657	1859	158	4.75
BARR	71.28258	-156.61874	T	Cold and dry	-12.5	29.4	183	72	134	102
BART	44.06388	-71.28731	TSF	Warm and wet	5.2	27.9	1262	545	362	8.5
BLAN	39.06026	-78.07164	WS	Warm and dry	12.2	24.4	954	-112	23	0
BONA	65.15505	-147.49100	BF	Cold and dry	-1.8	35.1	415	15	138	32.4
DCFS	47.16165	-99.10656	WS	Warm and dry	4.3	35.5	427	-303	57	0
DEJU	63.88112	-145.75136	BF	Cold and dry	-3.1	36.3	443	69	117	4.9
HARV	42.53690	-72.17266	TSF	Warm and wet	7.2	26.4	1113	350	211	7
HEAL	63.87569	-149.21334	BF	Cold and dry	-2.4	29.4	460	108	176	48.4
MLBS	37.37828	-80.52484	TSF	Warm and wet	8.9	22	1174	309	72	2.25
ORNL	35.96412	-84.28260	TSF	Warm and wet	13.9	22.4	1384	190	14	2.5
RMNP	40.27591	-105.54592	TSF	Warm and wet	2.4	20.8	704	55	328	0.63
SJER	37.10878	-119.73228	TGD	Warm and dry	16.1	19.7	469	-984	3	3
SOAP	37.03337	-119.26219	WS	Warm and dry	12.7	17.3	767	-408	15	2
STEI	45.50894	-89.58637	TSF	Warm and wet	4.7	31.9	806	104	150	2
TREE	45.49373	-89.58572	TSF	Warm and wet	4.7	31.9	807	105	149	2
WREF	45.82049	-121.95191	TRF	Warm and wet	8.9	17.7	2468	1630	255	4.67
CLBJ	33.40123	-97.57000	WS	Warm and dry	17.7	23.3	870	-524	1	0

Table S1 Continued

Site	Latitude	Longitude	Whittaker biome	Site climate group	MAT	Tdiff	MAP	MAP-Eref	PAS	Organic mat thickness
		Decimal °	Decimal °		°C	°C	mm	mm	mm	cm
CPER	40.81553	-104.74560	TGD	Warm and dry	8.2	25	348	-626	28	0
DELA	32.54172	-87.80389	TSF	Warm and wet	17.3	20.6	1403	25	2	0
DSNY	28.12504	-81.43620	TSF/S	Warm and wet	22.2	12.3	1282	-269	0	0
GRSM	35.68896	-83.50195	TSF	Warm and wet	12.1	20.2	1471	307	19	0
JORN	32.59068	-106.84254	TGD	Warm and dry	15.3	22	266	-1270	2	0
KONA	39.11044	-96.61295	WS	Warm and dry	12.5	29.1	854	-181	18	0
KONZ	39.10077	-96.56309	WS	Warm and dry	12.3	29.1	863	-157	20	0
LENO	31.85388	-88.16122	TSF	Warm and wet	18	19.4	1421	-41	1	0
MOAB	38.24833	-109.38827	TGD	Warm and dry	9.4	27.1	301	-755	32	0
NOGP	46.76972	-100.91535	WS	Warm and dry	5	34.4	406	-341	53	0
NIWO	40.05425	-105.58237	BF	Cold and dry	0.1	20.7	866	339	529	0
OAES	35.41059	-99.05879	WS	Warm and dry	15.7	25.9	699	-642	3	0
ONAQ	40.17759	-112.45244	TGD	Warm and dry	8.8	26.3	278	-727	38	0
OSBS	29.68927	-81.99343	TSFS	Warm and wet	20.5	14.9	1368	-192	0	0
SCBI	38.89292	-78.13950	TSF	Warm and wet	12	23.6	1015	-29	23	0
SERC	38.89008	-76.56001	TSF	Warm and wet	13.5	24.2	1076	34	15	0
SRER	31.91068	-110.83549	SD	Warm and dry	18.5	18.9	393	-1221	1	0
STER	40.46190	-103.02930	WS	Warm and dry	9.1	26.5	430	-561	22	0
TALL	32.95046	-87.39327	TSF	Warm and wet	16.9	20.7	1431	70	3	0
TOOL	68.66109	-149.37047	T	Cold and dry	-10.1	37.2	365	162	177	0
UNDE	46.23388	-89.53725	TSF	Warm and wet	4.3	31.5	815	130	173	0
WOOD	47.12823	-99.24136	WS	Warm and dry	4.3	35.3	420	-312	50	0
