# Supplementary material for: Climate and Ecosystem Factors Mediate Soil Freeze‐Thaw Cycles at the Continental Scale
Source: J Geophys Res Biogeosci. 2024 Nov 27;129(12):e2024JG008009. doi: 10.1029/2024JG008009 (PMC11600542; doi:10.1029/2024JG008009)
Supplement: Supplementary file 3 — Table S2 [file JGRG-129-0-s003.pdf]

Table S2. List of full site names.

Site ID	Full site name
ABBY	Abby Road
BARR	Utqiaġvik
BART	Bartlett Experimental Forest
BLAN	Blandy Experimental Farm
BONA	Caribou Poker
CLBJ	Caddo-Lyndon B. Johnson National Grasslands
CPER	Central Plains Experimental Range
DCFS	Dakota-Coteau
DEJU	Delta Junction
DELA	Dead Lake
DSNY	Disney
GRSM	Great Smokey Mountains National Park
HARV	Harvard Forest
HEAL	Healy
JORN	Jornada
KONA	Konza Agriculture
KONZ	Konza Core
LENO	Lenoir Landing
MLBS	Mountain Lake
MOAB	Moab
NIWO	Niwot Ridge
NOGP	Northern Great Plains Research Laboratory
OAES	Marvin Klemme Range Research Station
ONAQ	Onaqui-Ault Steppe
ORNL	Oak Ridge
OSBS	Ordway-Swisher
RMNP	Rocky Mountain National Park
SCBI	Smithsonian Conservation Biology Institute
SERC	Smithsonian Environmental Research Center
SJER	San Juaquin
SOAP	Soaproot Saddle
SRER	Santa Rita
STEI	Steigerwalt
STER	Sterling
TALL	Talladega
TOOL	Toolik
TREE	Treehaven

Table S2 continued.

Site ID	Full site name
UNDE	University of Notre Dame Environmental Research Center
WOOD	Woodworth
WREF	Wind River
