# Supplementary material for: Climate and Ecosystem Factors Mediate Soil Freeze‐Thaw Cycles at the Continental Scale
Source: J Geophys Res Biogeosci. 2024 Nov 27;129(12):e2024JG008009. doi: 10.1029/2024JG008009 (PMC11600542; doi:10.1029/2024JG008009)
Supplement: Supplementary file 4 — Table S3 [file JGRG-129-0-s002.pdf]

Table S3. Multiple linear regression model F-test (ANOVA) results. For each site or soil variable, model terms included the main effects of each variable, climate group (warm and wet, warm and dry, cold and dry), and the interaction between these terms on the maximum number of freeze-thaw cycles (FTC) within a given season. DF = degrees of freedom; SSE = sum of squared error; MSE = mean squared error. Adjusted R^2 applies to the overall multiple linear regression model.

Variable	Adjusted R^2	Season	Model term	DF	SSE	MSE	F-value	p-value
Tdiff	0.64	fall	Tdiff	1	2.06	2.06	7.9	0.018**
			Group	1	1.34	1.34	5.12	0.047**
			Interaction	1	3.42	3.42	13.12	0.005***
			Residuals	10	2.61	0.26	NA	NA
	-0.11	spring	Tdiff	1	0.21	0.21	0.13	0.726
		spring	Group	1	2.66	2.66	1.64	0.232
		spring	Interaction	1	0.08	0.08	0.05	0.826
		spring	Residuals	9	14.59	1.62	NA	NA
	0.56	winter	Tdiff	1	5.71	5.71	7.28	0.018**
		winter	Group	1	12.19	12.19	15.54	0.002***
		winter	Interaction	1	0.39	0.39	0.5	0.493
		winter	Residuals	13	10.2	0.78	NA	NA
MAP-Eref	0.38	fall	MAP-Eref	1	0.01	0.01	0.03	0.863
		fall	Group	1	4.46	4.46	9.85	0.011**
		fall	Interaction	1	0.43	0.43	0.96	0.350
		fall	Residuals	10	4.53	0.45	NA	NA
	0.20	spring	MAP-Eref	1	0.18	0.18	0.15	0.704
		spring	Group	1	5.94	5.94	5.04	0.051*
		spring	Interaction	1	0.84	0.84	0.72	0.420
		spring	Residuals	9	10.59	1.18	NA	NA
	0.27	winter	MAP-Eref	1	10.57	10.57	8.17	0.013**
		winter	Group	1	0.97	0.97	0.75	0.402
		winter	Interaction	1	0.12	0.12	0.09	0.770
		winter	Residuals	13	16.82	1.29	NA	NA

Table S3 continued.

Variable	Adjusted R^2	Season	Model term	DF	SSE	MSE	F-value	p-value
MAP	-0.07	fall	MAP	1	0.03	0.03	0.04	0.840
		fall	Group	1	0.9	0.9	1.16	0.307
		fall	Interaction	1	0.75	0.75	0.97	0.347
		fall	Residuals	10	7.75	0.77	NA	NA
	0.24	spring	MAP	1	5.08	5.08	4.58	0.061*
		spring	Group	1	1.95	1.95	1.75	0.218
		spring	Interaction	1	0.53	0.53	0.47	0.509
		spring	Residuals	9	9.99	1.11	NA	NA
	0.28	winter	MAP	1	5.68	5.68	4.46	0.055*
		winter	Group	1	4.29	4.29	3.36	0.090*
		winter	Interaction	1	1.95	1.95	1.53	0.238
		winter	Residuals	13	16.57	1.27	NA	NA
MAT	0.24	fall	MAT	1	0	0	0	0.989
		fall	Group	1	3.36	3.36	6.09	0.033**
		fall	Interaction	1	0.57	0.57	1.03	0.335
		fall	Residuals	10	5.51	0.55	NA	NA
	0.27	spring	MAT	1	2.44	2.44	2.3	0.164
		spring	Group	1	0.48	0.48	0.45	0.518
		spring	Interaction	1	5.08	5.08	4.78	0.056*
		spring	Residuals	9	9.55	1.06	NA	NA
	0.36	winter	MAT	1	5.19	5.19	4.58	0.052*
		winter	Group	1	7.36	7.36	6.5	0.024**
		winter	Interaction	1	1.19	1.19	1.05	0.324
		winter	Residuals	13	14.74	1.13	NA	NA

Table S3 continued.

Variable	Adjusted R^2	Season	Model term	DF	SSE	MSE	F-value	p-value
Organic mat	-0.06	fall	Organic mat	1	0.07	0.07	0.09	0.773
		fall	Group	1	0.89	0.89	1.16	0.304
		fall	Residuals	11	8.47	0.77	NA	NA
	0.45	spring	Organic mat	1	9.57	9.57	12	0.006**
		spring	Group	1	0	0	0	0.946
		spring	Residuals	10	7.98	0.8	NA	NA
	0.27	winter	Organic mat	1	6.17	6.17	4.77	0.046**
		winter	Group	1	4.23	4.23	3.27	0.092*
		winter	Residuals	14	18.09	1.29	NA	NA
PAS	0.31	fall	PAS	1	0.3	0.3	0.59	0.460
		fall	Group	1	0.59	0.59	1.19	0.301
		fall	Interaction	1	3.56	3.56	7.14	0.023**
		fall	Residuals	10	4.99	0.5	NA	NA
	0.02	spring	PAS	1	0.33	0.33	0.23	0.645
		spring	Group	1	3.3	3.3	2.29	0.164
		spring	Interaction	1	0.98	0.98	0.68	0.431
		spring	Residuals	9	12.95	1.44	NA	NA
	0.35	winter	PAS	1	10.08	10.08	8.77	0.011**
		winter	Group	1	2.77	2.77	2.41	0.145
		winter	Interaction	1	0.71	0.71	0.62	0.446
		winter	Residuals	13	14.94	1.15	NA	NA
